# Brain age gap as a diffusion MRI-based marker of traumatic brain injury-related brain changes and associated outcomes

**DOI:** 10.1093/braincomms/fcag254

**Published:** 2026-07-01

**Authors:** Livia Rodrigues, Drew Parker, Nima Broomand Lomer, Alexa E Walter, Daniel Brennan, Douglas H Smith, Jeffrey Ware, Andrea L C Schneider, Ramon Diaz-Arrastia, Ragini Verma

**Affiliations:** DiCIPHR Lab, Department of Radiology, University of Pennsylvania Perelman School of Medicine, Philadelphia, PA 19104, USA; DiCIPHR Lab, Department of Radiology, University of Pennsylvania Perelman School of Medicine, Philadelphia, PA 19104, USA; DiCIPHR Lab, Department of Radiology, University of Pennsylvania Perelman School of Medicine, Philadelphia, PA 19104, USA; Department of Neurology, University of Pennsylvania Perelman School of Medicine, Philadelphia, PA 19104, USA; Department of Neurology, University of Pennsylvania Perelman School of Medicine, Philadelphia, PA 19104, USA; Department of Neurosurgery, University of Pennsylvania Perelman School of Medicine, Philadelphia, PA 19104, USA; DiCIPHR Lab, Department of Radiology, University of Pennsylvania Perelman School of Medicine, Philadelphia, PA 19104, USA; Department of Neurology, University of Pennsylvania Perelman School of Medicine, Philadelphia, PA 19104, USA; Department of Biostatistics, Epidemiology, and Informatics, University of Pennsylvania Perelman School of Medicine, Philadelphia, PA 19104, USA; Department of Neurology, University of Pennsylvania Perelman School of Medicine, Philadelphia, PA 19104, USA; DiCIPHR Lab, Department of Radiology, University of Pennsylvania Perelman School of Medicine, Philadelphia, PA 19104, USA

**Keywords:** diffusion MRI, brain age gap, traumatic brain injury, age predictor, biomarker

## Abstract

Traumatic brain injury is a common neurological disorder and a leading cause of long-term disability, presenting with heterogeneous cognitive, emotional, and functional impairments. A critical clinical challenge is the early identification of patients at risk for persistent symptoms. The Brain Age Gap (BAG), the difference between an individual’s predicted brain age from imaging data and their chronological age, has emerged as a potential marker of injury-related brain changes. Here, we aim to evaluate diffusion-MRI-derived BAG for identifying traumatic brain injury patients (Glasgow Coma Scale 13–15) at risk for persistent symptoms. For this, we trained a normative age-prediction model on >13 000 healthy controls, and applied it to traumatic brain injury patients. We associated BAG clinical scores that evaluate processing speed and executive function (Trail Making Test Parts A and B), verbal memory (Rey Auditory Verbal Learning Test), general processing speed (Wechsler Adult Intelligence Scale), post-concussion symptoms (Rivermead Post-Concussion Symptoms Questionnaire), psychological distress (Brief Symptom Inventory-18) and insomnia severity (Insomnia Severity Index). Analyses included: (i) cross-sectional comparisons across three BAG-based subgroups: BAG+, BAGn and BAG−, representing patients with higher, neutral and lower BAGs relative to healthy controls, (ii) longitudinal linear mixed-effects models evaluating BAG measured at 2-week post-injury (BAG_2wk_) in symptom trajectories and (iii) prognostic logistic regression for prespecified poor 12-month outcomes. All statistical analyses were performed using Python libraries, including SciPy and Statsmodels. The age-prediction model demonstrated high accuracy and reliability (mean absolute error = 3.05 ± 3.67 years; intraclass correlation = 0.93). Cross-sectionally, higher BAG was associated with greater symptom burden. Compared with BAG− patients, those in the BAG+ subgroup reported higher Insomnia Severity Index (d = 0.411; *P* = 0.038) and Rivermead Post-Concussion Symptoms Questionnaire scores (d = 0.415; *P* = 0.038). Similarly, BAGn patients showed higher Brief Symptom Inventory (d = −0.419; *P* = 0.040), Insomnia Severity Index (d = −0.382; *P* = 0.038) and Rivermead Post-Concussion Symptoms Questionnaire (d = −0.525; *P* = 0.002) scores relative to BAG−. Longitudinally, higher BAG_2wk_ was associated with worse Rivermead Post-Concussion Symptoms Questionnaire (β = 0.195, 95% CI[0.093, 0.297]; partial R^2^ = 0.017; *P* = 0.0016) and worse Insomnia Severity Index (β = 0.107, 95% CI[0.033, 0.181]; partial R^2^ = 0.010; *P* = 0.0196) without time interaction. BAG_2wk_ modified change over time in Trail Making Test Parts A (β = 0.017, 95% CI 0.002–0.056; partial R^2^ = 0.043; *P* = 0.016). Finally, adding BAG_2wk_ into the prognostic model yielded a significant improvement in 12-month outcome prediction (likelihood-ratio test = 6.40; *P* = 0.011). Together, these findings indicate that diffusion-MRI-derived BAG is a robust and reproducible biomarker that captures clinically meaningful heterogeneity as early as two-week post-injury.

## Teaser

Rodrigues *et al*. present a diffusion-MRI-derived brain age gap as an early biomarker of clinical outcomes following traumatic brain injury. Elevated brain age gap measured 2-week post-injury was associated with greater symptom burden, poorer cognitive recovery, and more accurate prediction of 12-month post-injury cognitive outcome.

## Graphical Abstract

**Graphical Abstract:**
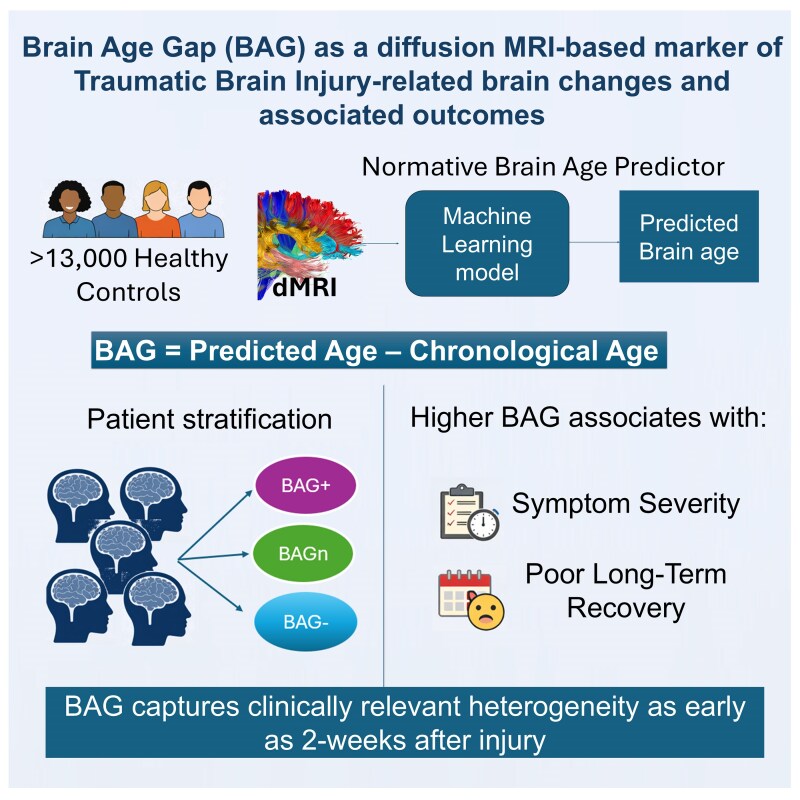

## Introduction

Traumatic brain injury (TBI) is a major cause of long-term disability worldwide.^1,2^ Outcomes are heterogeneous, spanning cognitive, emotional, and functional impairments that diminish quality of life,^3,4^ and the early identification of patients at risk for persistent symptoms remains challenging.^4^ The brain age gap (BAG), the difference between imaging-predicted brain age and chronological age, has emerged as a sensitive marker of injury-related deviation from normative aging trajectories.^5-10^ Elevated BAG may signal a more injured brain and, therefore, worse long-term outcomes after TBI and could serve as an early, individualized biomarker.

Structural MRI (sMRI) have been widely used to estimate BAG in TBI patients, with some studies reporting associations between BAG and poorer cognitive outcomes.^11,12^ However, imaging in these studies was typically performed months or even years after the injury,^11-13^ with some reporting correlation between BAG and time since injury.^11,12^ These findings suggest that brain atrophy following TBI evolves gradually,^12^ thereby limiting the utility of sMRI-derived BAG as an early prognostic biomarker.

TBI patients do not always present with lesions visible on CT or sMRI. This is particularly the case for individuals with Glasgow Coma Scale (GCS) scores of 13–15 and negative CT, who are frequently discharged from emergency or urgent care without further evaluation.^14,15^ However, many of these patients experience persistent cognitive and emotional symptoms for months to years after injury.^16-19^ These deficits have been linked to diffuse brain injuries such as diffuse axonal injury (DAI) and diffuse vascular injury(DVI)^20,21^ caused by shearing and tensile forces inducing differential motion across brain regions during rapid acceleration and deceleration of the brain, which cannot be adequately captured by conventional sMRI.

By contrast, diffusion MRI (dMRI) provides increased sensitivity to microstructural damage. dMRI-derived metrics, including fractional anisotropy (FA), mean diffusivity (MD), axial diffusivity (AD), and radial diffusivity (RD), capture distinct yet complementary features of white matter (WM) microstructure, and have proven valuable in detecting TBI-related abnormalities, including DAI and DVI.^22-28^ However, while these metrics are useful in group-level analyses comparing individuals with and without TBI, their interpretation at the individual level remains limited, posing a challenge for personalized prognosis.

A small number of studies have investigated dMRI-based BAG,^29,30^ with one study identifying significant correlations between BAG and information-processing speed in TBI patients months after injury.^29^ However, few studies have investigated the use of dMRI-based BAG for personalized prognostic assessment in TBI.

In this exploratory study, we developed a machine learning-based normative brain age predictor using dMRI-derived WM metrics from healthy controls (HCs) (ages 18–75) across four independent datasets. We then applied this model to TBI patients (GCS 13–15) to estimate their BAG. We first examined the dMRI-based BAG-defined subgroups using clinical scores to determine whether patients could be stratified into distinct groups. We then extended analysis to the full cohort, treating BAG as a continuous measure to model longitudinal trajectories of cognition and self-reported symptoms up to 12-month post-injury. Finally, we explore the prognostic utility of BAG alongside other demographical variables for predicting poor long-term outcomes, indicating that BAG could offer information relevant to individualized prognosis.

## Materials and methods

### Datasets

For creating the normative model that predicts brain age, we used HCs from four datasets: IXI (Information eXtraction from Images),^31^ UK Biobank (UKBB),^32^ TRACK-TBI (Transforming Research and Clinical Knowledge in TBI),^33^ and TBIRI (TBI Research Initiative), a locally recruited cohort at the University of Pennsylvania.

Both the TBIRI and TRACK-TBI^33^ studies were approved by the institutional review boards of the participating sites, and all participants, or their legally authorized representatives, provided written informed consent. The UKBB^32^ and IXI^31^ datasets are publicly available and were accessed in accordance with their respective data use policies.

For TBI data, we used the TRACK-TBI^33^ and TBIRI datasets. All patients presented with a GCS of 13–15 and had no trauma-related abnormalities on MRI, although we also analysed a subgroup with MRI-confirmed contusions (Supplementary Materials, Section S.1, Supplementary Fig. 1, and Supplementary Tables 1–3). In TRACK-TBI,^33^ TBI patients and HCs were scanned at 2-week and 6-month post-injury, while in TBIRI, TBI patients were scanned at the same intervals and HCs at a single timepoint. dMRI acquisitions details and image preprocessing are summarized in Supplementary Materials, Section S.2, Supplementary Table Supplementary Table 4.

For TRACK-TBI,^33^ clinical data including demographics and clinical scores were collected from individuals with TBI at 2-week, 6-month, and 12-month post-injury/post-enrolment. Cognitive performance was measured using the Trails Making Test Parts A and B (TMTA/TMTB),^34^ measuring processing speed and executive function (lower times indicates better performance), Rey Auditory Verbal Learning Test (RAVLT; Immediate and Delayed Recall),^35^ evaluating verbal memory (higher scores indicate better performance) and Wechsler Adult Intelligence Scale-IV Processing Speed Index (WAIS-PSI),^36^ assessing processing speed (higher scores indicate better performance). Self-reported symptom severity was assessed using somatic scores: the Rivermead Post-Concussion Symptoms Questionnaire (RPQ),^37^ which measures common post-concussion symptoms, Brief Symptom Inventory 18 (BSI),^38^ assessing psychological distress, and the Insomnia Severity Index (ISI),^39^ evaluating insomnia severity. For each of these three tests, higher scores indicate worse symptoms.

### Development of machine learning-based dMRI-based brain age predictor

#### Brain age prediction using normative modelling

To develop the normative brain age predictor, we first formed a training dataset including HC data from UKBB^32^ (45–75years, *n* = 13 199), IXI (20–75 years, *n* = 321) and 80% of TRACK-TBI^33^ dataset (18–71 years; 2-week post-enrolment, *n* = 73). Each subject was characterized by a feature vector that comprised 84 WM ROIs pertaining to FA, MD, AD and RD measures. Model performance was assessed using a hold-out set that included HCs from TBIRI and 20% of the TRACK-TBI^33^ cohort that were not used during training or validation, resulting in 66 HCs (ages 20–71 years).

Given the broad and imbalanced age distribution of our datasets (Table 1), with an overrepresentation of older adults from UKBB,^32^ and the nonlinear trajectory of brain maturation and aging,^29^ we segmented the age range to better capture developmental/aging trends and mitigate dataset imbalance. Therefore, we decided to train three independent models to capture age-specific brain patterns: Model 1 (18–35 years), Model 2 (30–50 years) and Model 3 (45–75 years). Each model used a combination of Support Vector Classifier and Lasso regression (Supplementary Materials, Section S.3.1, Supplementary Table Supplementary Table 6). Feature selection was optimized separately for each model and guided by a dedicated validation set, independent of the final hold-out data (Supplementary Table Supplementary Table 5).

**Table 1:** Demographic characteristics of the datasets included in this study

Set type	Dataset	*n*	Age range (years)	Male/female	Education (years)
Training/validation (HC only)	UKBB^32^	13 199	45–75	6153/7046	–
	IXI^31^	321	20–75	138/183	–
	TRACK-TBI^33^	109	18–71	72/37	6 (0–11 years) 63 (12–15 years) 39 (16+ years)
Hold-out (HC only)	TRACK-TBI^33^	20	20–59	11/9	2 (0–11 years) 12 (12–15 years) 6 (16+ years)
	TBIRI	46	20–71	29/17	–
TBI Patient	TRACK-TBI^33^	464	18–75	319/145	29 (0–11 years) 265 (12–15 years) 167 (16+ years)
	TBIRI	81	18–71	61/20	–

Education level information was not available for all participants.

During testing, the brain age of a participant was predicted by the model corresponding to their chronological age. For individuals whose age fell within the overlapping range of two models, the final prediction was calculated as the average of both model outputs (Supplementary Fig. 2). This overlapping strategy was implemented to reduce the age-related prediction bias at the extremes of the age distribution, which is frequently observed in regression models.^40,41^ While this approach decreases bias in the central age range, residual bias persisted at the extremes, with younger individuals tending to be predicted as older, and older individuals as younger. Given the lack of consensus on optimal bias correction methods,^42^ in all analyses, we used the raw BAG values and included chronological age as a covariate. To assess whether bias correction would meaningfully impact our results, we conducted a sensitivity analysis applying linear correction following.^42^

Model performance was quantified using Mean Absolute Error (MAE) and Pearson’s correlation coefficient (r) between predicted and chronological age.

#### Assessing normative model reliability

To evaluate the model’s reliability, we conducted a test–retest experiment using HCs from the TRACK-TBI^33^ hold-out set (*n* = 20, age range 20–59) who had imaging data acquired at two different timepoints, approximately 6 months apart. Brain age was predicted at each timepoint, and the MAE was calculated relative to the chronological age at each respective timepoint. To assess whether the prediction distributions remained stable between sessions, we used Intraclass Correlation Coefficient (ICC) and the Mann–Whitney U test. Given the short interval and the stable condition of HCs (Supplementary Materials, Section S.3.2), we did not expect meaningful changes in brain age estimates. Therefore, similar prediction distributions would support the model’s stability over time.

#### Brain age gap

The brain age predictor was then applied to TBI patient data from TRACK-TBI^33^ and TBIRI to estimate their brain ages, and to compute their BAG, defined as the difference between predicted age and chronological age. Since the TBI datasets (TRACK-TBI^33^ and TBIRI) contain data from two distinct timepoints, we calculated BAG at 2-week post-injury (BAG_2wk_) and 6-month post-injury (BAG_6mo_).

### Different use cases of brain age gap as a marker for prognosis

#### Cross-sectional comparison of BAG-based groups

TBI patients were categorized into three groups based on their BAG_tp_ relative to the standard deviation of HC (SD_HC_) BAG_tp_ predictions: BAG-Positive (BAG+; BAG_tp_>+1SD_HC_), BAG-Neutral (BAGn, −1SD_HC_ ≤ BAG_tp_ ≤ +1SD_HC_), or BAG-Negative (BAG−, BAG_tp_<−1SD_HC_), and we investigated the clinical significance of the groups by associating them with clinical and self-reported scores. The ±1SD_HC_ thresholds were selected to balance detection of clinically meaningful deviations with adequate subgroup sizes. Associations with clinical and self-reported outcomes were evaluated in cross-sectional analyses including participants with available data for each measure (Supplementary Tables 7 and 8).

For each outcome, two covariate-adjusted linear models were fitted: the first included age, sex and education (given their known associations with TBI outcomes^43-45^) and the second additionally incorporated BAG group as a factor, with HCs as reference.

#### Associations between BAG and longitudinal trajectories of clinical scores

We assessed whether BAG_2wk_ explained longitudinal variation using linear mixed-effects models fit separately for each clinical score. Fixed effects were Time Since Injury (days), age, sex, and education; continuous predictors (time, age, BAG) were z-scored. Models specified a participant-level random intercept. No random slopes were included. For each score, we fit three nested models: a covariate-only model (time, age, sex, education); a main-effect model adding BAG_2wk_ and an interaction model including BAG_2wk_ × (Time Since Injury).

This analysis was performed for each clinical score using data from three timepoints: 2-week, 6-month and 12-month post-injury. Because mixed-effects models are relatively robust to missing data, all patients with at least one available score at any timepoint were included. To reduce skewness, clinical scores that deviated from normality, as assessed by QQ-plots, were log-transformed prior to modelling.

#### BAG as a marker of poor 12-month outcome

We evaluated the long-term outcome prediction capability of BAG_2wk_. Our definition of poor outcome was based on those used in prior studies^46^ of the TRACK-TBI^33^ cohort. Cognitive decline and cognitive impairment for each patient was defined using five cognitive scores: TMTA, TMTB, RAVLT (Immediate and Delayed Recall) and WAIS. Cognitive Decline was determined using Reliable Change Index (RCI), by comparing a patient’s 12-month score to their highest pre-12-month performance, obtained either at the 2-week or 6-month timepoints. Cognitive Impairment was defined based on 12-month scores using normative ninth percentile thresholds tailored to each cognitive test. Normative baselines were defined using TRACK-TBI^33^ and TBIRI HC data (*n* = 66). Following the premises defined by,^46^ all tests were adjusted for age, and for the Cognitive Impairment analysis, TMTA and TMTA scores were additionally adjusted for sex, education, and race.^46^

Based on,^46^ we adopted three alternative definitions of 12-month poor cognitive outcome: (1) Cognitive Impairment or Cognitive Decline on at least one score on two or more tests, or a combination of both cognitive impairment and cognitive decline; (2) Cognitive Impairment or Cognitive Decline in two or more of the five scores and (3) Cognitive Impairment or Cognitive Decline in one or more of the five scores.

To assess long-term outcome based on self-reported symptomatic outcomes, we adapted a similar approach used for cognitive data using three sex and age-adjusted self-reported scores (RPQ, BSI and ISI). In this case, we define Symptom Worsening as determined by RCI (analogous to cognitive decline) and High Symptom Severity, based on HC (*n* = 66) ninth percentile thresholds at 12 months (analogous to Cognitive Impairment). Patients meeting criteria for any of the measures were classified as having a 12-month poor self-reported symptomatic outcome.

We tested whether BAG_2wk_ improved prediction of the outcome using nested multivariable logistic regression. The covariate-only model included age, sex, and education level while the full model additionally included BAG_2wk_.

We included only patients with complete data at all three time points: 192 for cognitive outcomes and 223 for self-reported outcomes.

### Statistical analysis

#### Cross-sectional comparison of BAG-based groups

We compared models with an F test to assess incremental variance explained by BAG group beyond demographics. When the overall group term was significant, we conducted prespecified pairwise contrasts among patient subgroups (BAG+ versus BAGn, BAG+ versus BAG−, BAG− versus BAGn) and versus HCs, reporting *P* values and Cohen’s d as effect size. Multiple comparisons within each time point were controlled using the Benjamini–Hochberg false discovery rate procedure (two-sided α = 0.05). Analyses were performed in Python using statsmodels and SciPy.

#### Associations between BAG and longitudinal trajectories of clinical scores

Likelihood-ratio tests compared (i) the main-effects model to the covariate-only model, (ii) the interaction model to the main-effects model and (iii) the interaction model to the covariate-only model to quantify the contribution of BAG_2wk_. Multiple testing was controlled with the Benjamini–Hochberg false discovery rate (two-sided α = 0.05). Effect sizes were summarized as partial R^2^, and model-based trajectories were generated from fixed-effect predictions at representative BAG levels (low, median and high) to illustrate between-participant differences in recovery.

#### BAG as a marker of poor 12-month outcome

The significance of $\mathrm{BA}G_{2wk}$ was assessed by comparing the full and reduced models using likelihood-ratio tests (LRTs). Discrimination was evaluated with the area under the receiver operating characteristic curve (AUC), and explanatory power was quantified using McFadden’s pseudo-R^2^ relative to a null intercept-only model, with ΔR^2^ reflecting the additional contribution of BAG. Regression coefficients were exponentiated to yield odds ratios (OR) with 95% confidence intervals, representing the multiplicative change in odds per unit increase in each predictor.

## Results

### Development of machine learning-based brain age predictor

#### Brain age prediction using normative modelling

The control hold-out set achieved a MAE of 3.05 ± 3.67 years (r = 0.955) between chronological and predicted age (Supplementary Fig. 4). In comparison, the TBI set produced a MAE of 4.32 ± 4.72 years (r = 0.953).

#### Assessing normative model reliability

For the subset of 20 HCs from TRACK-TBI,^33^ the MAE between 6-month chronological age and predicted age was 3.79 ± 4.38 years, and the MAE for 2-week chronological age and predicted age was 3.39 ± 4.17 years. There was no significant difference between BAG_2wk_ and BAG_6mo_ (ICC = 0.93; *P* = 0.73, Supplementary Fig. 5).

#### Brain age gap

**Figure 1:**
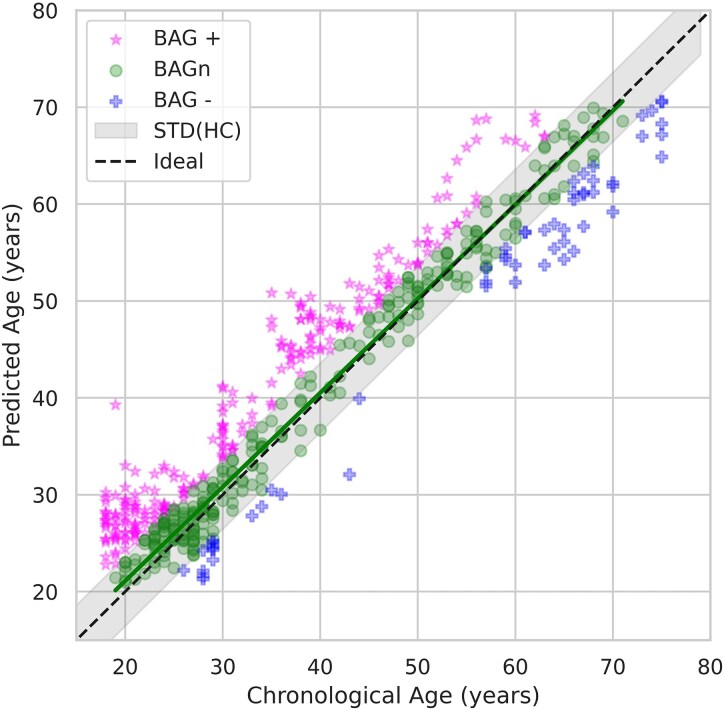
**Chronological age and predicted brain age of patients at the 2-week timepoint.** The dashed line represents a Brain Age Gap (BAG) = 0 for every age, and the grey band represents BAG values within 1 SDHC (standard deviation of Healthy Controls) among HCs from the line. Patients were categorized as BAG+ (red stars, *n* = 227) if BAG_2wk_ > +1 SD_HC_, BAGn (green circles, *n* = 265) if −1 SD_HC_ ≤ BAG_2wk_ ≤ +1 SD_HC_, or BAG− (blue cross, *n* = 53) if BAG_2wk_ < −1 SD_HC_.

When applying the model to all participants, our results showed that TBI patients present significant higher BAG when compared to HCs (median BAG = 2.62 for TBI versus 0.95 for HCs, *P* < 0.001, Supplementary Fig. 6). Then, TBI patients were stratified into three groups using their BAG_2wk_ values and found to have the following demographics: BAG+: *n* = 227, age 18–63 years, 28% female; BAGn: *n* = 265, age 19–71 years, 31% female; and BAG−: *n* = 53, age 26–75 years, 37.8% female (Fig. 1 and Supplementary Table 9).

Comparison of BAG_2wk_ and BAG_6mo_ in TBI patients revealed no significant difference (*P* = 0.27). Using BAG_6mo_ to define the groups, most TBI patients (78%) remained in the same group as defined by BAG_2wk_ (Table 2). Among 81 TBI patients who changed groups, only one moved from BAG− at 2-weeks to BAG+ at 6-months, and two moved from BAG+ at 2-weeks to BAG− at 6-months. In the last case, in which patients transitioned from BAG+ to BAG−, analysis of their scores across the two time points revealed improvements in TMTA, TMTB, WAIS, BSI and RPQ, alongside declines in RAVLT Delayed Recall and RAVLT Immediate Recall in both instances. One patient maintained their ISI score, whereas the other showed improvement (Supplementary Table 10). For the patient who transitioned from BAG− to BAG+, clinical scores were not available.

**Table 2:** Stability of BAG-based patient group classification over time

	BAG+ (2-weeks)	BAGn (2-weeks)	BAG− (2-weeks)
BAG+ (6-months)	123 (32.89%)	21 (5.61%)	1 (0.27%)
BAGn (6-months)	31 (8.29%)	141 (37.70%)	10 (2.67%)
BAG− (6-months)	2 (0.54%)	16 (4.28%)	29 (7.75%)

The table shows the number of patients classified as BAG+, BAGn, or BAG− at 2-week and 6-month post-injury.

As a sensitivity analysis, we applied linear bias correction following,^42^ using parameters estimated in HCs (α = 3.24, β = 0.93). The maximal change in BAG values was 2 years, with typical changes across the central age range being <1 year. All statistical results and conclusions remained unchanged.

### Different use cases of brain age gap as a marker for prognosis

#### Cross-sectional comparison of BAG-based groups

Statistically significant differences in clinical scores were observed between BAG_2wk_-based groups and HCs across multiple timepoints. When compared to HCs at 2-week post-injury, BAG+ TBI patients had worse BSI (d = 0.384; *P* = 0.022), RPQ (d = 0.780; *P* < 0.001), RAVLT Immediate Recall (d = −0.216; *P* = 0.031) and TMTA (d = 0.256; *P* = 0.022) scores. At 6 month post-injury, the BAG + group had worse BSI-18 (d = 0.411; *P* = 0.024), ISI (d = 0.444; *P* = 0.002) and RPQ (d = 0.698; *P* < 0.001) scores when compared to HCs, with worse RPQ even at 12-month post-injury (d = 0.472; *P* = 0.01). At 2-week post-injury, when compared to HCs, the BAGn group showed worse BSI (d = 0.430; *P* = 0.001) and RPQ (d = 0.880; *P* < 0.001), and also had worse RPQ scores at 12-month post-injury (d = 0.499; *P* = 0.01). The BAG group did not differ significantly from HCs at any timepoint (Supplementary Table 11).

When grouping TBI patients by BAG_6mo_, BAG+ group had worse ISI (d = 0.408; *P* = 0.023) and RPQ (d = 0.659; *P* = 0.008) scores when compared to HCs at 6-months. BAGn presented worse BSI (d = 0.452; *P* = 0.008) and RPQ (d = 0.777; *P* < 0.001) scores when compared to HCs at 6-months. BAG− group did not differ significantly from HCs at any timepoint. No significant difference was found between BAG_6mo_-based subgroups and HCs at 12-month post-injury (Supplementary Table 11).

We also conducted direct comparisons of clinical scores between TBI patient subgroups. Using BAG_2wk_, statistically significant differences emerged in 2-week clinical scores: BAG+ had worse ISI and RPQ scores than BAG− (d = 0.411; *P* = 0.038 and d = 0.415; *P* = 0.038, respectively), and BAGn reported worse BSI (d = −0.419; *P* = 0.04), ISI (d = −0.382; *P* = 0.038) and RPQ (d = −0.525; *P* = 0.002) scores than BAG−. At 6-month post-injury, BAG+ had worse RPQ scores than BAG− (d = 0.432; *P* = 0.007) and BAGn had worse RPQ scores when compared to BAG− (d = −0.469; *P* = 0.007). No differences were found in 12-month clinical scores. BAG_6mo_-based stratification did not show any significant differences in clinical scores (Supplementary Table 12).

Individual correlations between BAG_2wk_ and BAG_6mo_ and the clinical scores are presented in the Supplementary Materials (Supplementary Figs 7 and 8).

### Associations between BAG and longitudinal trajectories of clinical scores

**Figure 2:**
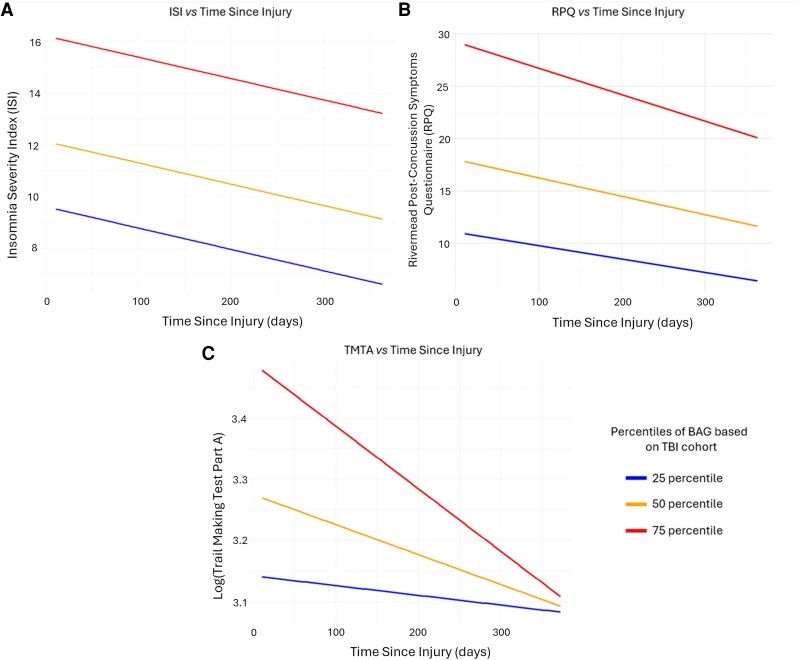
**Model-predicted trajectories.** (**A**) Insomnia Severity Index (ISI), (**B**) Rivermead Post-Concussion Symptoms Questionnaire (RPQ), and (**C**) Trail Making Test Part A (TMTA) scores over time based on BAG values from the TBI cohort. Curves represent the predicted mean clinical scores at the 25th, 50th, and 75th percentiles of BAG (−0.28, 2.52, and 5.59, respectively). Time since injury (days) is shown on the *X*-axis, and clinical scores on the *Y*-axis. Linear mixed-effects models with random intercepts for participants were used, adjusting for age, sex, and education. Higher BAG values were consistently associated with worsening ISI and RPQ scores over time (likelihood-ratio test partial R^2^ = 0.017, *P* = 0.0016, and partial R^2^ = 0.010, *P* = 0.0196, respectively). For TMTA, the effect of BAG was time-dependent (BAG × time interaction: likelihood-ratio test, partial R^2^ = 0.043, *P* = 0.016), indicating that individuals with higher BAG initially performed worse but improved over time. Models were trained with 192 patients.

Linear mixed-effects models showed that higher BAG_2wk_ values were associated with worse RPQ (β = 0.195, 95% CI[0.093, 0.297], partial R^2^ = 0.017, *P* = 0.0016) and ISI (β = 0.107, 95% CI[0.033, 0.181], partial R^2^ = 0.010, *P* = 0.0196) scores throughout the entire follow-up period, with no significant interaction between BAG_2wk_ and time since injury. Thus, patients with higher BAG_2wk_ values started with and maintained worse RPQ and ISI when compared to those with lower BAG_2wk_. A significant BAG_2wk_ and Time since injury interaction was found for TMTA (β = 0.017, 95% CI[0.002, 0.056], partial R^2^ = 0.043, *P* = 0.016), (Fig. 2), whereby individuals with higher BAG_2wk_ values exhibited worse initial TMTA but showed greater improvement over time, with trajectories converging towards those with lower BAG_2wk_. No significant main or interaction effects of BAG_2wk_ were observed for BSI, RAVLT (Immediate or Delayed Recall), TMTA or WAIS scores (Supplementary Table 13).

Additional interaction terms were explored, and results are provided in the Supplementary Materials (Supplementary Materials, Section S.7.2 and Supplementary Fig. 9).

### BAG as a marker of 12-month outcome

**Figure 3:**
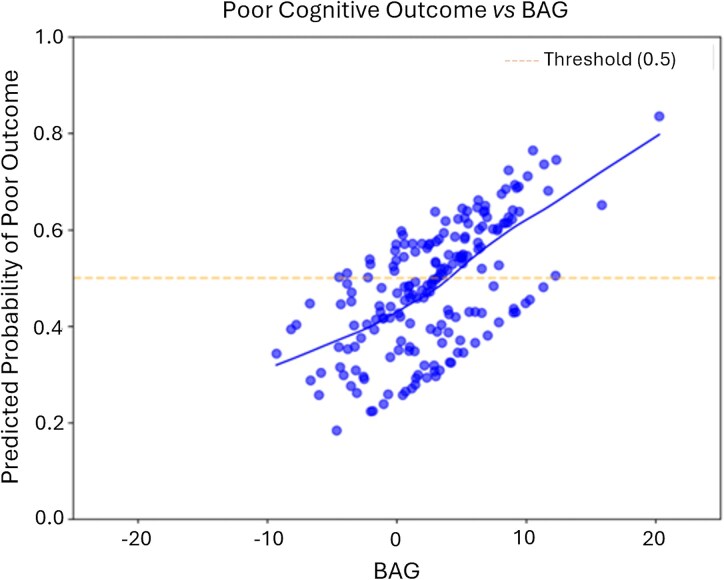
**Brain Age Gap at 2 weeks (BAG_2wk_) versus the probability of poor cognitive outcome.** Here, we use Definition 2 of poor cognitive outcome (Cognitive Impairment or Cognitive Decline in two or more of the five scores). The association was evaluated using logistic regression (*N* = 192 patients). Each datapoint represents one participant. Higher BAG_2wk_ was associated with greater odds of poor cognitive outcome (odds ratio = 1.09, 95% CI: 1.02–1.18, *P* = 0.018). The line indicates the 50% probability threshold.

Of the three definitions of cognitive outcome assessed, BAG_2wk_ significantly predicted poor outcome under Definition 2 (Fig. 3). Inclusion of BAG_2wk_ significantly improved model fit over the reduced model without BAG_2wk_ (LRT = 6.40, *P* = 0.011). Discrimination increased from an AUC of 0.603 to 0.665 (ΔAUC = 0.062), and McFadden’s pseudo-R^2^ improved from −0.334 to −0.301 (ΔR^2^ = 0.033). BAG_2wk_ was independently associated with cognitive outcome in the fully adjusted model (OR = 1.09, 95% CI[1.02, 1.18], *P* = 0.018). For Definitions 1 and 3 for cognitive outcome, no significant effects of BAG_2wk_ were observed. The same held true for self-reported outcomes (Supplementary Table 14).

In an exploratory analysis, we examined decline and impairment outcomes for individual clinical scores. BAG_2wk_ significantly predicted decline in TMTA and BSI, with a marginal effect for TMTA. It also significantly predicted impairment in ISI (Supplementary Table 15). Additional models including BAG-by-demographic interaction terms revealed no significant effects (Supplementary Materials, Section S.7.3).

## Discussion

In this study we built a dMRI-based brain age model using HCs, validated its ability to track chronological aging and then applied that model to a cohort of TBI patients (GCS 13–15, MRI-negative for contusions) to test whether early deviations in estimated brain age, the brain age gap (BAG), carry prognostic information beyond routine demographic and clinical measures.

We first confirmed that the predictor captured a biologically meaningful signal. The model estimated chronological age with low error (MAE ≈ 3 years; SD_HC_ ≈ 3.7 years), implying that small deviations within ±1 SD_HC_ likely reflect statistical variability, whereas larger BAG deviations are more plausibly indicative of altered brain microstructure. We assessed the reproducibility of model in a subset of HCs who underwent repeated scans over a 6-month interval. The test–retest analyses returned high ICC (0.93), confirming that BAG estimates in HC are highly stable over 6 months. This stability indicates that the changes observed in patients are unlikely to represent measurement artefacts but instead reflect genuine biological or clinically relevant processes. Given that, in accordance with prior studies,^11,12,29,30^ our results showed that patients with TBI exhibited elevated BAG compared with HCs, supporting the interpretation that BAG may capture deviations from normative brain organization, potentially reflecting injury-related microstructural alterations.

When patients were stratified into BAG_2wk_ and BAG_6mo_ groups, the majority (∼78%) remained in the same BAG category across timepoints. Only 22% transitioned between groups, most commonly from BAG+ to BAGn or from BAGn to BAG−. Transitions from BAG− to BAG+ and from BAG+ to BAG− were rare. Shifts towards more favourable groups, particularly when substantial (e.g. BAG+ to BAG−), may reflect microstructural recovery and corresponding clinical improvement. In our sample, two patients who shifted from BAG+ to BAG− also showed marked improvements on TMTA, TMTA, BSI, ISI and RPQ at 6 months. However, the limited sample size (*n* = 2) restricts the strength of this conclusion.

It is important to notice that the observed stability of dMRI-based BAG over the 6-month follow-up suggests no evidence of ongoing accelerated brain aging in this period. While this finding may initially appear to contrast with prior structural MRI studies in TBI reporting associations between brain age and time since injury,^12^ these observations are not necessarily contradictory. Structural MRI-based brain age measures may be more sensitive to slower, cumulative neurodegenerative or atrophic processes, whereas dMRI-derived BAG likely captures early, diffuse microstructural alterations that emerge rapidly after injury and then plateau.

After estimating brain age in patients and deriving BAG_2wk_ and BAG_6mo_ values, we conducted two complementary sets of analyses focusing on individual scores (cross-sectional and longitudinal) and an additional set of analyses that aggregated scores to define broader cognitive and self-reported 12-month outcomes. Our aim was to understand how BAG relates to each score/outcome across different timepoints.

The results of these approaches were complementary and mutually reinforcing. For self-reported scores, cross-sectional analyses showed that BAG+ and BAGn groups, defined using BAG_2wk_, reported worse RPQ, BSI, and ISI scores than HCs at one or more timepoints, with RPQ consistently worse across all timepoints. Similarly, BAG+ and BAGn patients performed worse than BAG− patients, again with RPQ remaining elevated up to 6-month post-injury. These findings were consistent with the longitudinal analyses, in which higher BAG_2wk_ reliably predicted worse RPQ and ISI scores across time, indicating sustained vulnerability to post-concussive and sleep-related symptoms.

For cognitive scores, only the BAG+ group differed significantly from HCs, showing worse TMTA and RAVLT Immediate Recall only at the 2-week timepoint. Longitudinal analyses aligned with these findings: a significant (BAG_2wk_ × Time Since Injury) interaction for TMTA indicated that patients with elevated BAG performed worse initially but gradually converged with lower-BAG individuals by 12-month. Direct comparisons between BAG+ and BAGn groups did not yield significant differences, a null result that may reflect either genuinely similar cognitive and symptom profiles or limited statistical power to detect moderate subgroup differences.

Analyses based on BAG_6mo_ continued to show differences between patient groups and HCs, as well as among patient subgroups, but these effects were attenuated relative to BAG_2wk_. This may be due in part to smaller sample sizes available for BAG_6mo_ analyses, but they may also reflect the greater prognostic value of BAG_2wk_, consistent with the interpretation that dMRI-derived BAG is most sensitive in the subacute post-injury phase.^25^

Our results on cognitive scores align with earlier findings. For example, higher sMRI-based BAG in TBI patients has been associated with worse performance on TMTA and TMTB,^11^ and dMRI-based BAG has been related to poorer information-processing speed as assessed by the Digit Symbol Coding test.^29^ Taken together, these converging results suggest that dMRI-derived BAG primarily reflects heightened vulnerability in processing speed and executive function, rather than memory performance or broad cognitive impairment. In contrast, we did not observe significant associations between BAG and WAIS scores. This may reflect the fact that WAIS captures distributed and multimodal brain functions, relying on both grey matter volume and white matter integrity,^47^ which could reduce its sensitivity to a global white matter–based measure like BAG. Consistently, BAG derived from T1-weighted images, which primarily reflect volumetric or intensity-based differences rather than microstructural integrity, has also shown weak associations with WAIS performance.^12^ Time elapsed since injury may further influence these patterns. For example, measures such as TMTA may be particularly sensitive to early microstructural changes, potentially driven by neuroinflammatory processes in regions including the frontostriatal system, corpus callosum, and cingulum.^48^ whereas broader composites like WAIS may better reflect long-term atrophic changes. Together, these considerations help explain why dMRI-derived BAG appears most closely linked to processing speed and executive function, rather than global cognitive measures.

Beyond individual tests, we also evaluated the predictive value of BAG for aggregated cognitive outcomes, using three operational definitions: (1) impairment on ≥2 tests across multiple domains (broad, cross-domain deficit); (2) impairment on two tests irrespective of domain (potentially more sensitive to multi-score deficits within a single domain) and (3) impairment on any one of five tests (a more permissive definition). BAG_2wk_ was associated only with Definition 2, suggesting that BAG predicts vulnerability within specific domains rather than a global deficit. This interpretation was supported by test-level analyses (Supplementary Table 10), which showed significant associations with TMTA, marginal effects for TMTA, and no associations with memory-related measures (RAVLT Immediate or Declined Recall) or WAIS subtests.

Finally, BAG_2wk_ did not associate with the aggregated self-reported outcome (ISI, BSI and RPQ), despite showing consistent associations with each measure individually in cross-sectional and longitudinal analyses. A likely explanation is that combining instruments with differing psychometric properties increased measurement noise and diluted specific effects, thereby reducing the sensitivity of the composite outcome to the signals captured by BAG.

The limitations of this study should be acknowledged. First and most important, the absence of pre-injury imaging limits our ability to distinguish between pre-existing brain characteristics and those resulting from TBI. As a result, a patient presenting with a neutral or negative BAG shortly after injury may still have experienced injury-related decline if their pre-injury brain age was lower than expected. This complicates the interpretation of BAG, as observed deviations may reflect differences in brain and cognitive reserve^49^ rather than injury effects. Consequently, elevated BAG values may reflect pre-morbid characteristics associated with increased TBI risk, such as prior head injuries, substance use, or socioeconomic factors influencing brain health, rather than injury-related effects per se. For example, previous studies have shown that higher BAG values are associated with worse sleep patterns,^50^ independent of health status, suggesting that BAG may reflect broader vulnerability rather than injury-specific effects. Additionally, although all HC used for model training were screened for neurological conditions, prior TBI history could not be fully excluded. Moreover, while BAG provides a global measure that may capture patterns similar to brain age, TBI is a highly heterogeneous condition. The relatively small size of our TBI cohorts limits the ability to fully account for this variability, underscoring the importance of external validation in larger, independent datasets. Finally, although BAG showed consistent associations with symptom burden and cognitive performance, effect sizes were modest, indicating limited prognostic value on its own. Rather than serving as a standalone marker, BAG may enhance prognostic models when integrated with clinical, demographic, and multimodal imaging measures. Despite these limitations, our results converge on a consistent message: dMRI-based BAG is a reproducible marker that captures clinically relevant heterogeneity as early as 2-weeks after injury, suggesting that follow-up may often be necessary for patients with GCS 13–15. Future studies could explore the feasibility and added value of acquiring imaging data even earlier than the 2-week timepoint. Also, future work should build on this work by incorporating advanced diffusion MRI models with greater biological specificity, such as neurite orientation dispersion and density imaging (NODDI) and free-water imaging, when supported by the study design and acquisition protocols. In parallel, future studies could consider calculating BAG for independent modules formed by regions specifically associated with individual cognitive or symptom domains, rather than relying on a single global metric. This modular approach may enable more individualized analyses and better capture the heterogeneity of patient symptoms and vulnerabilities. In addition, the prognostic value of BAG should be evaluated alongside other biomarkers (e.g. blood-based measures), demographic factors, including socioeconomic variables, and complementary imaging metrics, to develop integrated multimodal models that maximize predictive accuracy and support early risk stratification in TBI. Translating BAG into clinical practice will require larger, independent datasets with MRI and clinical data collected both before and after injury, in order to establish sensitivity, specificity, predictive values, and clinically actionable thresholds while accounting for outcome prevalence. Such studies are also needed to assess real-world utility, including practical considerations regarding imaging timing and feasibility in routine clinical settings.

## Supplementary Material

fcag254_Supplementary_Data

## Data Availability

The statistical analysis code is available at https://github.com/diciphr-lab. TRACK-TBI data are available on FITBIR (https://fitbir.nih.gov/). The harmonized and processed data can be made available on contacting the corresponding author and following the appropriate regulatory process for TRACK-TBI and the ancillary studies using their data.
