# Supplementary material for: Brain age gap as a diffusion MRI-based marker of traumatic brain injury-related brain changes and associated outcomes
Source: Brain Commun. 2026 Jul 1;8(4):fcag254. doi: 10.1093/braincomms/fcag254 (PMC13353526; doi:10.1093/braincomms/fcag254)
Supplement: fcag254_Supplementary_Data [file fcag254_supplementary_data.pdf]

Supplementary Materials

S.1 Patients with Contusions

Of the original 545 patients, 82 presented sMRI findings consistent with contusion. As our primary analyses focused on patients without such findings, these individuals were excluded from the main experiments. However, for completeness, we report here the results obtained from the full patient cohort, including those with contusions.

S.1.1 Cross-sectional comparison of BAG-based groups

When comparing patient subgroups with HCs and within themselves, we observed that all clinical scores showing significant differences were consistent with those identified in the analysis excluding patients with contusion (Supplementary Table 1).

Supplementary Table 1: *Significant Results in BAG2wk-based group analysis of Clinical Scores when adding patients with contusions*

	2-weeks (corrected p-val; d)	6-months (corrected p-val; d)	12-months (corrected p-val; d)
HC vs BAG+	BSI(0.022, 0.385)<br>RAVLT-Immediate(0.031 -0.216)<br>RPQ(0.0, 0.78)<br>TMTA(0.022, 0.256)	BSI(0.024, 0.411)<br>ISI(0.002, 0.444)<br>RPQ(0.0, 0.698)	RPQ(0.010, 0.472)
HC vs BAG_n	BSI(0.001, 0.43)<br>RPQ(0.0, 0.879)	RPQ(0.0 0.742)	RPQ(0.010, 0.499)
BAG+ vs BAG-	ISI(0.038, 0.411)<br>RPQ(0.038, 0.415)	RPQ(0.007, 0.432)	--
BAG_n vs BAG-	BSI(0.04, -0.419)<br>ISI(0.038, -0.382)<br>RPQ(0.002, -0.525)	RPQ(0.007 -0.469)	--

S.1.2 Associations between BAG and Longitudinal Trajectories of Clinical Scores

In the longitudinal analysis of clinical scores (Supplementary Table 2), the BSI reached statistical significance when contusion cases were included (p = 0.0404), whereas it had shown only a trend in the original analysis, without the patients with contusion (p = 0.0691).

Supplementary Table 2: *Significant Results in Longitudinal analysis of Clinical Scores when adding patients with contusions*

	**Corrected p-value**	**Partial R^2 (BAG)**	**Partial R^2 (BAG*TSI – IM)**	**β [95%CI]**
**TMTA**	0.0058	0.0068	0.0031	0.0389 [-0.005, 0.083]
**RPQ**	0.0058	0.0117	0.2016	0.131 [-0.019, 0.281]
**ISI**	0.0296	0.0081	0.8257	0.013 [-0.094, 0.120]
**BSI**	0.0404	0.0049	0.1188	0.036 [-0.090, 0.162]

S.1.3 BAG as a marker of *12-months* outcome

The analysis on poor 12-months outcome returned similar results when we added the patients with contusion (Supplementary Table 3): BAG_{2wk} showed modest incremental value only for Definition 2 of cognitive outcomes (LRT = 4.566, p = 0.033), with slight improvements in discrimination (ΔAUC = 0.054) and model fit (ΔMcFadden R^2 = 0.020). For the other cognitive outcome definitions and self-reported symptoms, BAG_{2wk} did not significantly improve predictive performance.

Supplementary Table 3: *Incremental value of BAG for different outcome definitions and self-reported symptoms.*

Outcome Definition	LRT (p)	ΔAUC	ΔMcFadden R^2	BAG OR [95% CI]
**Definition 1**	1.024 (0.312)	0.020	0.005	1.042 [0.969–1.121]
**Definition 2**	1.810 (0.179)	0.019	0.007	1.043 [0.981–1.109]
**Definition 3**	**4.566 (0.033)**	**0.054**	**0.020**	**1.073 [1.004–1.147]**
**Self-reported symptoms**	2.743 (0.098)	0.023	0.008	1.049 [0.991–1.110]

S.1.4 How dMRI-based BAG is affected in patients with contusion

Together, the presented findings suggest that BAG, being derived from dMRI, may be unaffected by the presence of contusions, unless these injuries significantly disrupt white matter microstructural integrity. The only difference emerged in the longitudinal BSI analysis, which became significant after including patients with contusions. However, since the initial result already showed a trend (corrected p-value = 0.0691), it is possible that the observed significance reflects the larger sample size, which allowed for a more robust statistical analysis.

To further support this hypothesis, we examined the individual BAG values of the 82 patients with contusions. All 84 ROI measures were corrected for age and sex in both HCs and patients. Normative standard deviations (STD_{HC}) were calculated from the control group data. For each patient, we quantified the number of ROIs considered impaired, defined as values exceeding +1.96 STD_{HC} (representing 95th percentile cut) for MD maps and falling below −1.96 STD_{HC} for FA maps. The same procedure was applied to clinical scores, with thresholds adjusted according to the directionality of each scale (e.g., lower scores indicating better performance on TMTA, and higher scores indicating better performance on WAIS).

As shown on Supplementary Figure 1, even though these patients exhibited visible findings on structural imaging, BAG values were associated with the number of ROIs deviating from the normative range. This is expected, given that brain age estimation is based on the dMRI data.

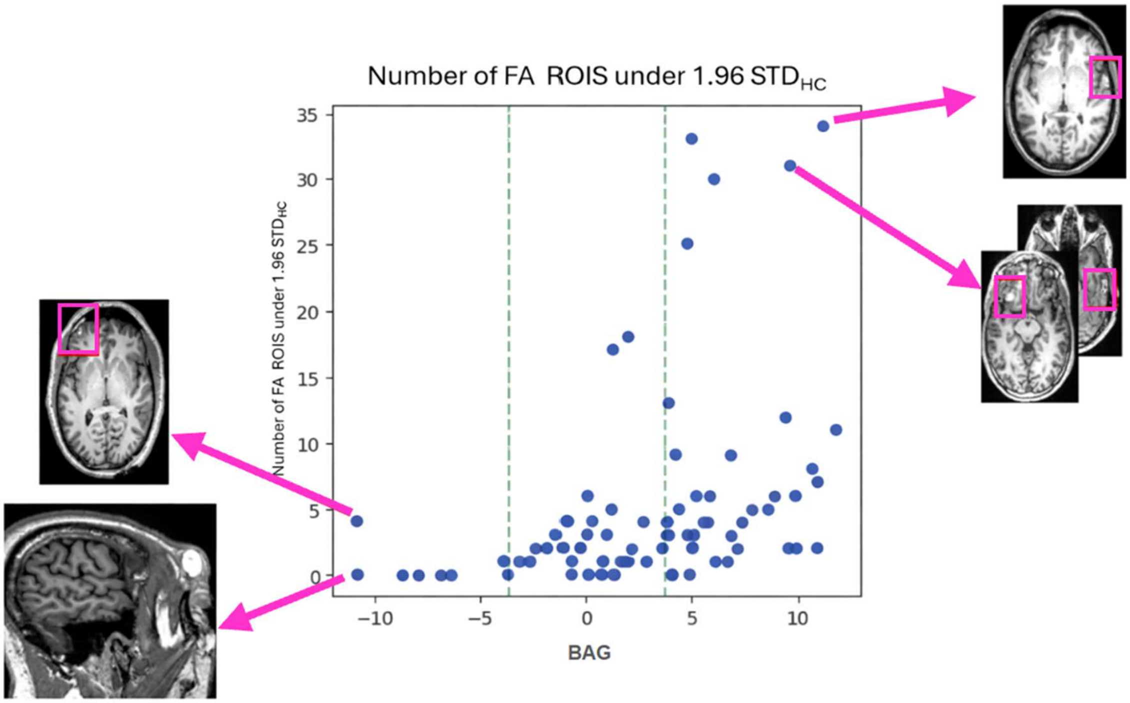

Supplementary Figure 1: *Relation between number of FA ROIs out of the normative range vs BAG on patients with contusion. As can be seen, the higher the BAG, the more the patients present ROIs out of the normative range.*

S.1.5 Conclusions

Interestingly, the predictive value of BAG remained consistent when analyses were restricted to patients without focal lesions on sMRI. This finding reinforces the hypothesis that BAG captures diffuse microstructural abnormalities that may not be apparent on standard MRI or CT, highlighting its potential to detect clinically relevant brain changes even in patients with negative imaging findings. Such sensitivity reinforces the utility of BAG as an integrative biomarker of brain health post-injury, capable of informing prognosis beyond what can be inferred from visible structural damage alone. This has important clinical implications, especially in TBI patients with GCS 13-15, where conventional imaging often fails to explain persistent symptoms or long-term cognitive decline.

S.2 Preprocessing and Harmonization

dMRI acquisitions details and image processing varied across datasets and are summarized in Supplementary Table 4. TRACK-TBI, IXI and UK Biobank (UKBB) were multi-site studies with their dMRI sequences coordinated to be as close as possible given scanner hardware available. TRACK-TBI dMRI originated from 11 sites, while IXI dMRI data came from 2 sites, and UKBB dMRI data was collected at 4 imaging sites[1].

T1 and dMRI data from TRACK-TBI, TBIRI, and IXI datasets were pre-processed with our in-house pipeline. T1 images were rigidly registered to the SRI24 atlas[2] with ANTs[3], where brain masks were estimated using BrainMaGe[4], a deep-learning based skull-striping method which was trained to handle pathological anatomy including brain tumors and contusions.

Supplementary Table 4: *Diffusion MRI acquisition details of the four data sources used.*

Dataset	Field Strength	Scanner Manufac-turer(s)	In plane resol-ution (mm)	Slice thick-ness (mm)	Image dimen-sions	Non-zero b-value	Grad-ient direc-tions	TR (ms)	TE (ms)	Head Coil
**TRACK-TBI**	3T	Siemens, GE, Philips	2.4x2.4 or 2.7x2.7	2.4 or 2.7	varies	1000 or 1300	64	3700 - 15500	70 - 110	32-channel
**TBIRI**	3T	Siemens Magnetom Prisma Fit	2.4x2.4	2.4	96x96x63	1000	64	2900	94	32-channel
**IXI**	1.5T or 3T	Philips	1.75x1.75 or 1.83x1.83	2 or 2.35	128x128x56 or 128x128x64	1000	15	9054 - 11895	51 - 80	Not listed
UKBB	3T	Siemens Skyra	2.0x2.0	2	104x104x72	1000 and 2000	50 per shell	3600	92	32-channel

Estimated brain masks were subsequently registered back to native space. dMRI scans were denoised using a local PCA method[5], followed by FSL's *eddy*[6] for simultaneous motion and eddy current correction. A brain mask was estimated using FSL's BET tool[7], and tensors were fit within the mask with a weighted least squares tensor-fitting method implemented in DIPY[8], after which fractional anisotropy (FA), mean diffusivity (MD) and axial (AD) and radial diffusivity (RD) were calculated. The average B0 image was extracted from the diffusion MRI data, which was registered to the T1 image in ANTs in three stages: first, the T1 was rigidly registered to the B0 image; a deformation field was estimated with the SyN algorithm[9] restricted to find displacements within the axial plane, accounting for susceptibility distortions induced within the axial slice by single-shot echo planar imaging; finally, the deformation field was applied to the B0 image, and a rigid registration was estimated to the T1 image.

dMRI data from UKBB were processed by the pipeline described in[10], and T1 data were processed by the pipeline described in[11], resulting in defaced T1 images and brain masks. Similarly to our in-house pipeline, the UKBB diffusion MRI pipeline included FSL's top-up[12] and eddy for susceptibility distortion correction and motion and eddy current correction, with differences in noise estimation methods, and including gradient distortion correction and Gibbs unwringing. The b=0 and b=1000 volumes were extracted from the diffusion dataset, a tensor was fit by DIPY's weighted least-squares method, and FA, MD, RD and AD were calculated. dMRI scan was registered to the T1 image with a rigid transformation estimated in ANTs.

Following the preprocessing steps, the same registration steps were applied to bring white matter regions of interest (ROIs) to dMRI space and calculate ROI statistics of FA, MD, AD and RD. Skull-stripped T1 images were registered to the JHU-MNI-Single Subject ("Eve") atlas[13] using a three-stage registration algorithm including rigid registration, followed by affine registration and deformation estimated by the SyN algorithm. The inverse of this deformation, along with the inverse of the affine transformation, and the inverse of the DTI-to-T1 transformations were composed, resulting in a transformation from the Eve atlas to diffusion MRI

space. Label maps were created in diffusion space by nearest neighbor interpolation, and 84 WM regions were selected from 102 WM regions in the Eve White Matter Parcellation Map[14]. Because IXI diffusion scans consistently acquired with the field of view cutting off the cerebellum, brainstem and midbrain, 14 regions including cerebellar WM, cerebellar peduncles, medial lemniscus and a portion of corticospinal tract were excluded. 4 more regions were excluded from analysis (bilateral fornix and tapetum) because they are often affected by partial volume and small inaccuracies in registration due to their small size and their location near the lateral ventricles.

The median values of FA, MD, AD and RD were calculated within the 84 selected WM ROIs. ROI-based median diffusion scalar values (2-weeks and 6-months post-injury/post-enrollment) were harmonized using ComBat[15] to correct for site-related variability, with age (continuous years), sex (male, female) and group (TBI, HC) included as covariates. Scans from the same study but different scanning sites were treated as originating from separate sites. Harmonization was performed separately at each timepoint to account for temporal differences, using the largest UKBB site as the reference batch in both cases.

S.3 Development of Machine Learning-based Brain Age Predictor

S.3.1 Brain Age Prediction using normative modeling

Our final model was composed of 3 independent models, each one focused on a different age range: Model 1 (18–35 years), Model 2 (30–50 years), and Model 3 (45–75 years). The specifics for each model can be found on Supplementary Table 5. Feature selection was guided by the performance on each model's validation set. We evaluated both forward selection (using Lasso) and PCA (with variance thresholds of 0.90 and 0.95) and selected the strategy that achieved the best results for each model.

To decide which maps we should use for each model, we ran the PCA several times using different combinations (FA, MD, FA+MD, FA+MD+AD, FA+MD+RD and FA+MD+AD+RD). The forward selection was done using all maps. All the experiments focused on finding the best model were done in the training set and using the validation set to find the best parameters. To prevent data leakage, the hold-out set was not used during this process.

As result, for Model 1 and Model 2 we applied PCA and for Model 3, we applied forward selection. The selected ROIS used in Model 3 are shown in Supplementary Table 6.

Given the small training dataset in the lower age ranges (Model 1 and Model 3), we applied synthetic data augmentation by averaging data from subjects of the same age and sex to generate new synthetic samples.

Supplementary Table 5: *Models details*

	Age Range (mean years)	Sex distribution (Male/Female)	Feature selection strategy	Diffusion maps used	Datasets used for training	Augmentation	Training set after augmentation (if used)
Model 1	18-35 (24.9)	61.3% / 38.7%	PCA (variance, 0.9)	FA and MD	TRACK / IXI	Yes	172
Model 2	30-50 (46.5)	48% / 52%	PCA (variance, 0.95)	FA, MD, AD	TRACK / IXI / UKBB	Yes	300
Model 3	45-75 (62.1)	47% / 53%	Forward selection	FA, MD, AD, RD	UKBB	No	11413

Supplementary Table 6: *ROIs used for Model 3 after applying Forward Selection*

FA	MD	AD	RAD
Posterior Limb of Internal Capsule Left,<br>Anterior Corona Radiata Left,<br>Fornix (Cres) / Stria Terminalis Left,<br>External Capsule Left,<br>Uncinate Fasciculus Left,<br>Retrolenticular Part of Internal Capsule Left,<br>Cingulum WM Left,<br>Fusiform WM Left,<br>Middle Occipital WM Left,<br>Superior Temporal WM Left,<br>Lateral Fronto-Orbital WM Left,<br>Genu of Corpus Callosum Right,<br>Inferior Occipital WM Right,<br>Rectus WM Right	Cerebral Peduncle Left,<br>Posterior Thalamic Radiation (Include Optic Radiation) Left	Cerebral Peduncle Left,<br>Anterior Limb of Internal Capsule Left,<br>Inferior Fronto-Occipital Fasciculus Left,<br>Anterior Limb of Internal Capsule Right,<br>Cingulum (Hippocampus) Right,<br>Superior Fronto-Occipital Fasciculus Right,<br>Splenium of Corpus Callosum Right	Posterior Limb of Internal Capsule Left,<br>Superior Corona Radiata Left,<br>Posterior Corona Radiata Left,<br>External Capsule Left,<br>Uncinate Fasciculus Left,<br>Cerebral Peduncle Right,<br>Superior Longitudinal Fasciculus Right

During inference, each subject was assigned to the model corresponding to their chronological age. For individuals whose age fell within the overlapping range of two models, the final prediction was calculated as the average of both outputs (Supplementary Figure 2). This overlapping strategy not only smooths the transition between models but also mitigates potential bias arising from the age imbalance in Model 3, where older participants (50+ years) are overrepresented.

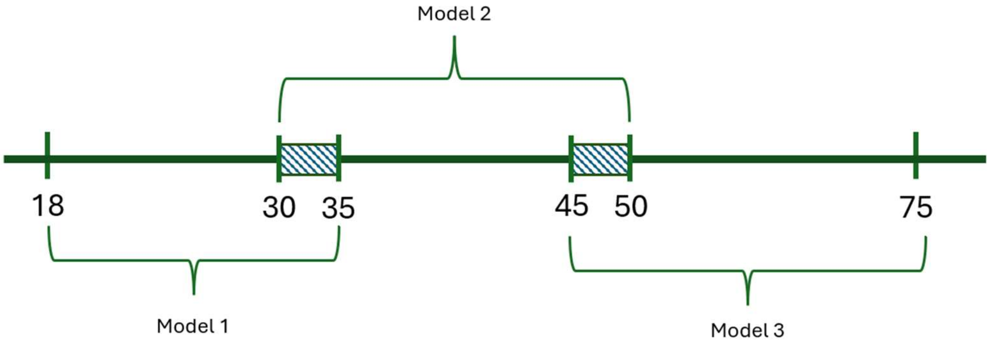

Supplementary Figure 2: *Inference schematics. Each Model focus on a different age range: Model 1 (18–35 years), Model 2 (30–50 years), and Model 3 (45–75 years)*

S.3.2 Assessing Normative Model Reliability

For test–retest reliability, predictions were assessed at two timepoints using 20 healthy controls (HCs) from the TRACK-TBI cohort. We assessed the HCs stability by comparing the 2-week and 6-months post-enrollment clinical scores (Supplementary Figure 3). In all cases, there was no significant difference between the two timepoints.

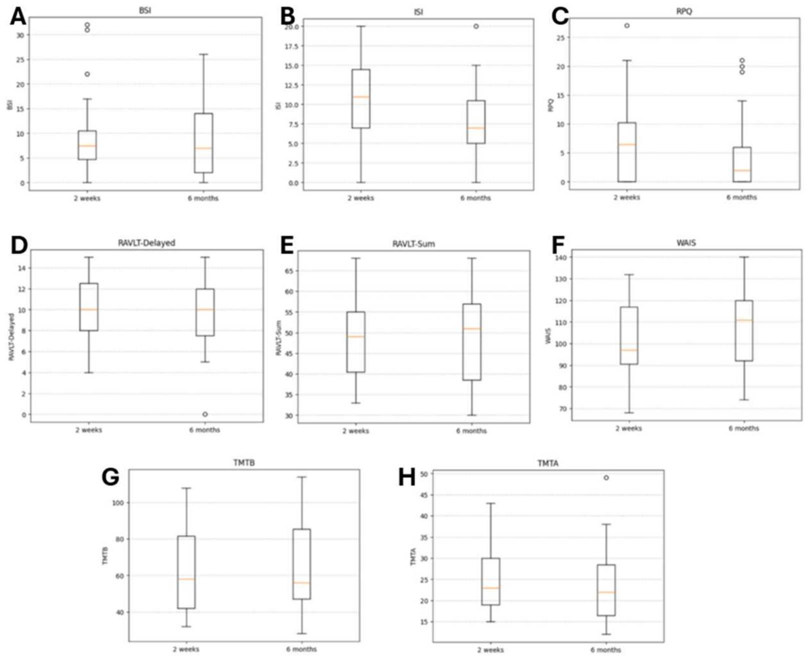

Supplementary Figure 3: *Clinical Scores from health controls used in reliability analysis (n=20). For all cases, there is no significant differences in paired t-test(p-val>0.05). A: Brief Symptom Inventory 18 (BSI); B: Insomnia Severity Index (ISI), C: the Rivermead Post-Concussion Symptoms Questionnaire (RPQ), D: Rey Auditory Verbal Learning Test (RAVLT Delayed Recall), E: RAVLT Immediate Recall), F: Wechsler Adult Intelligence Scale-IV Processing Speed Index (WAIS), G: Trail Making Test Part B (TMTB), H:Trail Making Test Part A (TMTA)*

S.4 Dataset

For the development of the brain age predictor, we used UKBB, IXI, and 80% of TRACK-TBI for training/validation. The hold-out set was composed of HCs from TBIRI and the remaining 20% from TRACK-TBI.

When combining patients from the TRACK-TBI and TBiRIs cohorts, a total of 545 patients with imaging data were available. Of these, 82 patients presented imaging findings indicative of contusions and were therefore excluded from the main study, however analysis including these patients is presented in Section S.1.

Among the HC cohort (from TRACK-TBI and TBiRi) and the remaining 463 patients, not all subjects had complete clinical data across all evaluated timepoints. Therefore, for the cross-sectional analyses, we included only those participants who had available data for the specific clinical score being assessed (Tables S7 and S8).

In contrast, for the longitudinal analyses, all available patients were included, as the mixture model employed is robust to missing data.

All patients used in the analysis have GCS (13-15).

Supplementary Table 7: *Number of patients used for cross sectional analysis*

	Score	2 weeks	6 months	12 months
**BAG_{2wk}**	**BSI**	357	289	236
	**ISI**	351	324	276
	**RAVLT-Delayed**	372	372	293
	**RAVLT-Immediate**	376	374	295
	**RPQ**	332	257	208
	**TMTA**	375	373	293
	**TMTB**	374	370	292
	**WAIS**	375	374	294
**BAG_{6mo}**	**BSI**	-	271	230
	**ISI**	-	271	230
	**RAVLT-Delayed**	-	269	208
	**RAVLT-Immediate**	-	269	209
	**RPQ**	-	271	232
	**TMTA**	-	268	207
	**TMTB**	-	266	207
	**WAIS**	-	269	208

Supplementary Table 8: *Number of HCs used for cross sectional analysis*

Score	2 weeks	6 months	12 months
**BSI**	109	107	94
**ISI**	108	107	95
**RAVLT-Delayed**	104	102	89
**RAVLT-Immediate**	105	103	89
**RPQ**	109	108	95
**TMTA**	103	103	88
**TMTB**	103	103	88
**WAIS**	103	102	88

S.5. Development of Machine Learning-based Brain Age Predictor

S.5.1 Brain Age Prediction using normative modeling

While several established approaches (e.g., Gaussian process regression, spline-based methods, and deep learning models) can capture nonlinear age trajectories within a unified framework, in our data we observed that a single model trained across the full age range resulted in higher prediction error (MAE ≈ 5 years), likely reflecting residual age imbalance and heterogeneous trajectories across the lifespan.

We therefore adopted an age-stratified approach to better account for distinct developmental and aging patterns, consistent with prior work[16]. The resulting models showed improved and comparable performance across age ranges (M1: MAE = 3.107; M2: MAE = 3.135; M3: MAE = 2.537), with the lower error in M3 likely reflecting its larger training sample (n = 13,199).

Importantly, the choice of three age ranges reflects a balance between model specificity and data availability. The younger groups already have relatively limited training samples (Supplementary Table 5), and further subdivision would reduce model stability. Also, although the older group has a larger training set, splitting it would substantially reduce the number of healthy controls from TRACK-TBI available for testing in each subgroup. We therefore selected three age ranges as a practical compromise between capturing nonlinear effects, maintaining stable training, and preserving adequate test sample sizes.

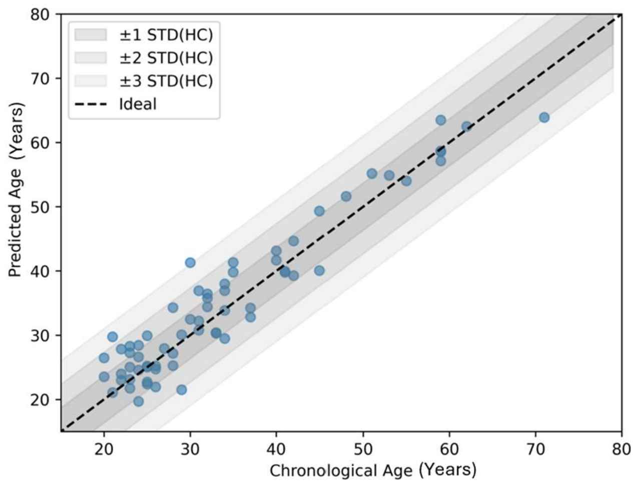

Supplementary Figure 4: *Each datapoint represent predicted and chronological age from HCs at 2-week timepoint data (n=66, Mean Absolute Error=3.05±3.67 years; Pearson's correlation: r=0.955). The gray bands (Standard Deviation of HC- SDHC) represent different standard deviation intervals relative to the healthy control group*

S.5.2 Assessing Normative Model Reliability

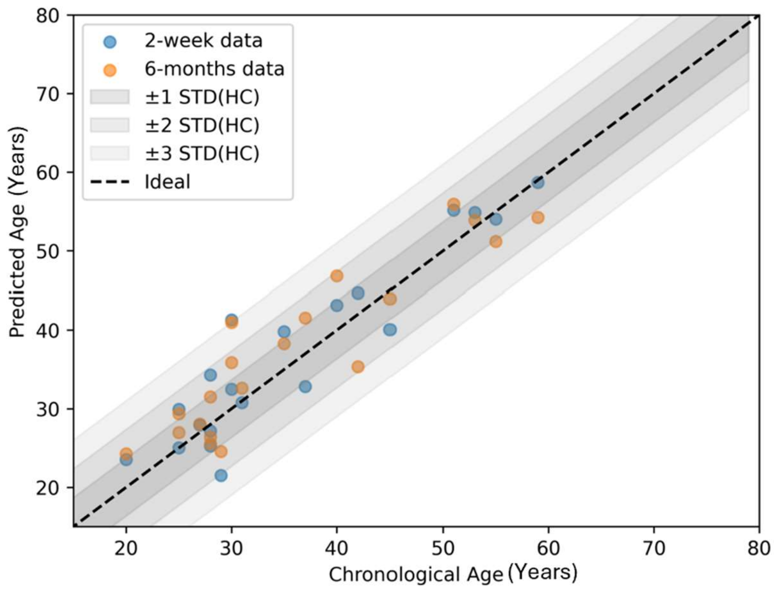

Supplementary Figure 5: *Each datapoint represent predictions at 2-week and 6-month vs chronological age (Healthy Controls, n=20, ICC=0.93; paired t-test p=0.73). The gray bands (SD_{HC}) represent different standard deviation intervals relative to the healthy control group*

S.6. Brain Age Gap

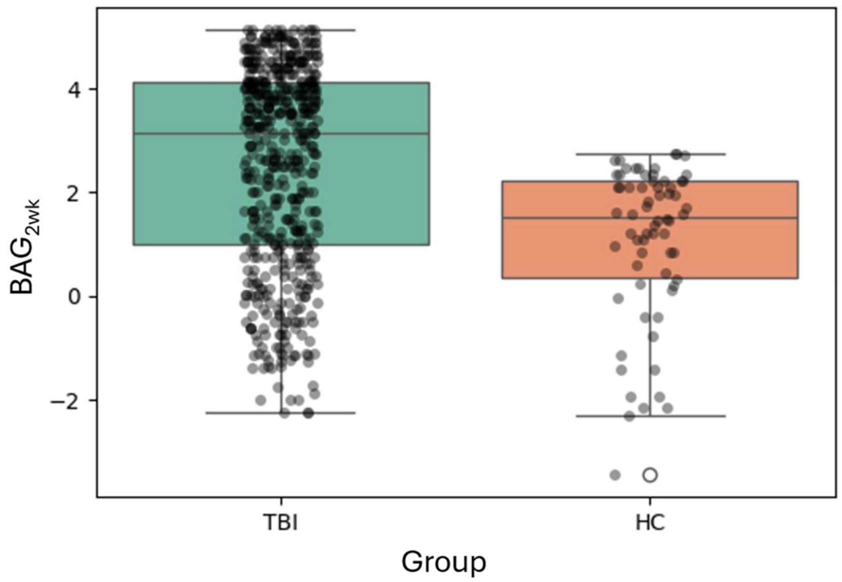

Supplementary Figure 6: *Comparison between TBI patients (n=545) and health controls (HC, n=66) Brain Age Gap (BAG), corrected for age and sex. Each datapoint represents one participant. TBI patients show a significant higher BAG (median BAG=2.62 for TBI vs. 0.95 for HCs, t-test p<0.001).*

Supplementary Table 9: *BAG2wk groups*

Group (n)	Age range	%Female
BAG+ (227)	18-63	28
BAGn(265)	19-71	31
BAG- (53)	26-75	37.8

Supplementary Table 10: *Scores for patients that shifted from BAG+ to BAG- groups after 6 months.*

Subject	Timepoint	TMTA	TMTB	RAVLT-S	RAVLT-D	WAIS	BSI	ISI	RPQ
S1	2-weeks	27	51	47	10	100	18	1	16
	6-months	21	39	43	7	114	6	1	0
S2	2-weeks	18	63	55	9	86	6	5	4
	6-months	15	49	53	7	97	0	0	0

S.7 Different Use Cases of Brain Age Gap as a Marker for Prognosis

For completeness, we present here all the results found in all analyses, including the non-significant ones. In bold, we highlight the statistically significant results after FDR correction.

S.7.1 Cross-sectional comparison of BAG-based groups

Supplementary Table 11: *BAG-based group vs HCs. Significant findings are in bold.*

		2 weeks (p; d)	6 months (p; d)	12 months (p; d)
**BAG_{2wk}-based stratification**	**BAG+ vs HC**	**BSI (0.022; 0.385)**<br>ISI (0.690; 0.061)<br>RAVLT-Delayed (0.234; -0.088)<br>**RAVLT-Immediate (0.031; -0.216)**<br>**RPQ (0.000; 0.780)**<br>**TMTA (0.022; 0.256)**<br>TMTB (0.456; 0.100)<br>WAIS (0.769; -0.043)	**BSI (0.024; 0.411)**<br>**ISI (0.002; 0.444)**<br>RAVLT-Delayed (0.753; -0.032)<br>RAVLT-Immediate (0.640; -0.077)<br>**RPQ (0.000; 0.698)**<br>TMTA (0.408; 0.082)<br>TMTB (1.000; -0.001)<br>WAIS (1.000; -0.034)	BSI (0.261; 0.279)<br>ISI (0.659; 0.086)<br>RAVLT-Delayed (0.487; -0.142)<br>RAVLT-Immediate (0.487; -0.163)<br>**RPQ (0.010; 0.472)**<br>TMTA (0.123; 0.184)<br>TMTB (0.563; 0.074)<br>WAIS (0.677; -0.095)
	**BAGn vs HC**	**BSI (0.001; 0.430)**<br>ISI (0.748; 0.043)<br>RAVLT-Delayed (0.515; -0.123)<br>RAVLT-Immediate (0.227; -0.184)<br>**RPQ (0.000; 0.879)**<br>TMTA (0.485; 0.146)<br>TMTB (0.772; 0.043)<br>WAIS (0.861; 0.048)	BSI (0.061; 0.311)<br>ISI (0.105; 0.245)<br>RAVLT-Delayed (0.444; -0.146)<br>RAVLT-Immediate (0.366; -0.174)<br>**RPQ (0.000; 0.742)**<br>TMTA (1.000; 0.041)<br>TMTB (1.000; 0.021)<br>WAIS (1.000; -0.020)	BSI (0.489; 0.144)<br>ISI (0.736; 0.039)<br>RAVLT-Delayed(0.188; -0.234)<br>RAVLT-Immediate (0.188; -0.221)<br>**RPQ (0.010; 0.499)**<br>TMTA (0.471; 0.173)<br>TMTB (0.188; 0.259)<br>WAIS (0.423; -0.158)
	**BAG- vs HC**	BSI (0.983; -0.040)<br>ISI (0.075; -0.410)<br>RAVLT-Delayed (0.690; -0.325)<br>RAVLT-Immediate (0.690; -0.503)<br>RPQ (0.558; 0.408)<br>TMTA (0.485; 0.558)<br>TMTB (0.769; 0.414)<br>WAIS (0.485; 0.041)	BSI (0.859; -0.201)<br>ISI (1.000; 0.167)<br>RAVLT-Delayed (0.640; -0.518)<br>RAVLT-Immediate (0.408; -0.553)<br>RPQ (1.000; 0.345)<br>TMTA (0.529; 0.254)<br>TMTB (1.000; 0.333)<br>WAIS (0.408; 0.046)	BSI (0.702; -0.022)<br>ISI (0.128; -0.289)<br>RAVLT-Delayed(0.822; -0.525)<br>RAVLT-Immediate (0.702; -0.554)<br>RPQ (0.940; 0.260)<br>TMTA (0.702; 0.763)<br>TMTB (0.563; 0.665)<br>WAIS (0.822; -0.295)
**BAG_{6mo}-based stratification**	**BAG+ vs HC**		BSI (0.326; 0.352)<br>**ISI (0.023; 0.408)**<br>RAVLT-Delayed (0.804; -0.013)<br>RAVLT-Immediate (0.326; -0.121)<br>**RPQ (0.008; 0.659)**<br>TMTA (0.523; 0.038)<br>TMTB (0.869; -0.043)<br>WAIS (0.869; -0.043)	BSI (0.390; 0.235)<br>ISI (0.767; 0.037)<br>RAVLT-Delayed (0.390; -0.144)<br>RAVLT-Immediate (0.390; -0.210)<br>RPQ (0.071; 0.515)<br>TMTA (0.390; 0.022)<br>TMTB (0.681; 0.030)<br>WAIS (0.716; -0.089)
	**BAGn vs HC**		**BSI (0.008; 0.452)**<br>ISI (0.064; 0.324)<br>RAVLT-Delayed (0.869; -0.083)<br>RAVLT-Immediate (0.869; -0.086)<br>**RPQ (0.000; 0.777)**<br>TMTA (0.869; 0.017)<br>TMTB (0.869; 0.021)<br>WAIS (0.869; -0.012)	BSI (0.390; 0.210)<br>ISI (0.631; 0.129)<br>RAVLT-Delayed (0.681; -0.177)<br>RAVLT-Immediate (0.681; -0.199)<br>RPQ (0.205; 0.428)<br>TMTA (0.716; 0.136)<br>TMTB (0.390; 0.257)<br>WAIS (0.716; -0.155)

	BAG- vs HC		BSI (0.869; -0.058)<br>ISI (0.869; 0.048)<br>RAVLT-Delayed (0.093; -0.727)<br>RAVLT-Immediate (0.179; -0.626)<br>RPQ (0.804; 0.501)<br>TMTA (0.869; 0.291)<br>TMTB (0.869; 0.210)<br>WAIS (0.845; -0.016)	BSI (0.849; 0.124)<br>ISI (0.390; -0.075)<br>RAVLT-Delayed (0.390; -0.734)<br>RAVLT-Immediate (0.390; -0.804)<br>RPQ (0.390; 0.626)<br>TMTA (0.631; 0.811)<br>TMTB (0.390; 0.814)<br>WAIS (0.767; -0.409)

Supplementary Table 12: *BAG-based group comparison. Significant findings are in bold*

		2 weeks (p; d)	6 months (p; d)	12 months (p; d)
**BAG_{2wk}-based stratification**	**BAG+ vs BAGn**	BSI (0.432; -0.047)<br>ISI (0.892; 0.015)<br>RAVLT-Delayed (0.587; 0.037)<br>RAVLT-Immediate (0.432; -0.026)<br>RPQ (0.361; -0.131)<br>TMTA (0.142; 0.120)<br>TMTB (0.432; 0.051)<br>WAIS (0.587; -0.089)	BSI (0.878; 0.122)<br>ISI (0.228; 0.198)<br>RAVLT-Delayed (0.840; 0.119)<br>RAVLT-Immediate (0.696; 0.106)<br>RPQ (0.946; -0.024)<br>TMTA (0.355; 0.043)<br>TMTB (0.889; -0.023)<br>WAIS (0.946; -0.015)	BSI (0.677; 0.123)<br>ISI (0.828; 0.048)<br>RAVLT-Delayed (0.677; 0.098)<br>RAVLT-Immediate (0.677; 0.078)<br>RPQ (0.828; 0.009)<br>TMTA (0.530; 0.015)<br>TMTB (0.564; -0.183)<br>WAIS (0.677; 0.056)
	**BAG+ vs BAG-**	BSI (0.192; 0.377)<br>**ISI (0.038; 0.411)**<br>RAVLT-Delayed (0.192; 0.252)<br>RAVLT-Immediate (0.432; 0.299)<br>**RPQ (0.038; 0.415)**<br>TMTA (0.587; -0.290)<br>TMTB (0.729; -0.319)<br>WAIS (0.361; -0.080)	BSI (0.124; 0.495)<br>ISI (0.136; 0.268)<br>RAVLT-Delayed (0.889; 0.521)<br>RAVLT-Immediate (0.696; 0.534)<br>**RPQ (0.007; 0.432)**<br>TMTA (0.137; -0.171)<br>TMTB (0.903; -0.358)<br>WAIS (0.291; -0.077)	BSI (0.437; 0.285)<br>ISI (0.060; 0.359)<br>RAVLT-Delayed (0.801; 0.376)<br>RAVLT-Immediate (0.872; 0.452)<br>RPQ (0.060; 0.288)<br>TMTA (0.677; -0.510)<br>TMTB (0.828; -0.604)<br>WAIS (0.677; 0.185)
	**BAG- vs BAGn**	**BSI (0.040; -0.419)**<br>**ISI (0.038; -0.382)**<br>RAVLT-Delayed (0.361; -0.211)<br>RAVLT-Immediate (0.729; -0.315)<br>**RPQ (0.002; -0.525)**<br>TMTA (0.729; 0.431)<br>TMTB (0.892; 0.348)<br>WAIS (0.455; -0.006)	BSI (0.136; -0.423)<br>ISI (0.372; -0.080)<br>RAVLT-Delayed (0.946; -0.383)<br>RAVLT-Immediate (0.889; -0.405)<br>**RPQ (0.007; -0.469)**<br>TMTA (0.360; 0.222)<br>TMTB (0.946; 0.328)<br>WAIS (0.256; 0.068)	BSI (0.564; -0.156)<br>ISI (0.060; -0.317)<br>RAVLT-Delayed(0.677; -0.263)<br>RAVLT-Immediate (0.748; -0.327)<br>RPQ (0.060; -0.304)<br>TMTA (0.833; 0.545)<br>TMTB (0.828; 0.440)<br>WAIS (0.564; -0.140)
**BAG_{6mo}-based stratification**	**BAG+ vs BAGn**		BSI (0.311; -0.141)<br>ISI (0.633; 0.071)<br>RAVLT-Delayed (0.633; 0.073)<br>RAVLT-Immediate (0.330; -0.039)<br>RPQ (0.370; -0.200)<br>TMTA (0.311; 0.022)<br>TMTB (0.677; -0.064)	BSI (0.995; 0.020)<br>ISI (0.860; -0.088)<br>RAVLT-Delayed (0.860; 0.037)<br>RAVLT-Immediate (0.860; 0.001)<br>RPQ (0.860; 0.108)<br>TMTA (0.860; -0.112)<br>TMTB (0.860; -0.231)

			WAIS (0.633; -0.032)	WAIS (0.995; 0.062)
	**BAG+ vs BAG-**		BSI (0.603; 0.357)<br>ISI (0.261; 0.335)<br>RAVLT-Delayed (0.311; 0.780)<br>RAVLT-Immediate (0.603; 0.557)<br>RPQ (0.311; 0.221)<br>TMTA (0.363; -0.255)<br>TMTB (0.633; -0.266)<br>WAIS (0.603; -0.027)	BSI (0.860; 0.120)<br>ISI (0.860; 0.107)<br>RAVLT-Delayed (0.860; 0.583)<br>RAVLT-Immediate (0.860; 0.645)<br>RPQ (0.860; -0.003)<br>TMTA (0.995; -0.747)<br>TMTB (0.860; -0.800)<br>WAIS (0.995; 0.312)
	**BAG- vs BAGn**		BSI (0.270; -0.438)<br>ISI (0.270; -0.261)<br>RAVLT-Delayed (0.261; -0.667)<br>RAVLT-Immediate (0.301; -0.601)<br>RPQ (0.261; -0.387)<br>TMTA (0.685; 0.286)<br>TMTB (0.731; 0.189)<br>WAIS (0.677; -0.004)	BSI (0.860; -0.098)<br>ISI (0.611; -0.199)<br>RAVLT-Delayed (0.860; -0.527)<br>RAVLT-Immediate (0.860; -0.588)<br>RPQ (0.995; 0.117)<br>TMTA (0.860; 0.598)<br>TMTB (0.860; 0.550)<br>WAIS (0.995; -0.265)

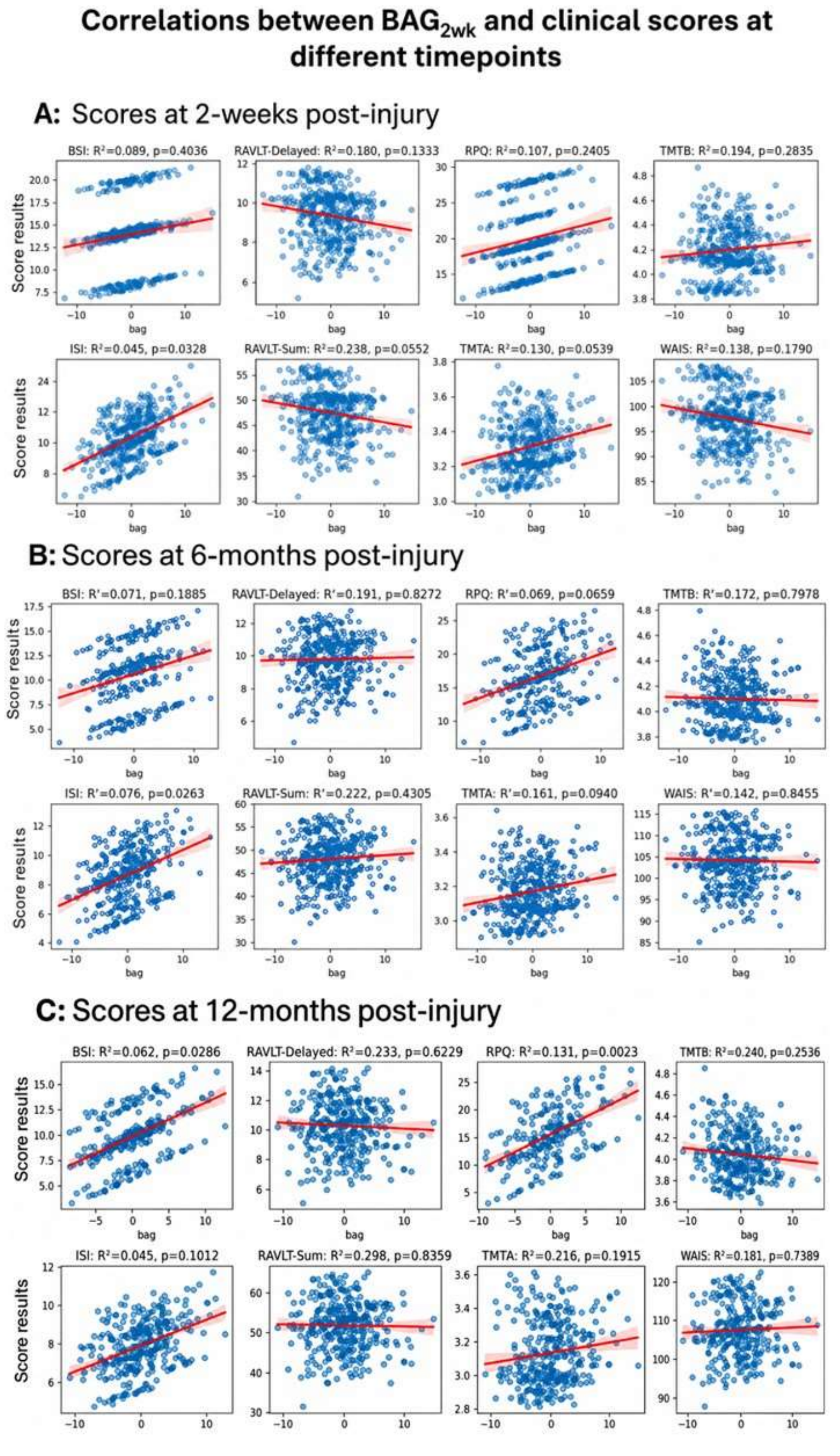

***Supplementary Figure 7**: Correlations between Brain Age Gap at 2 weeks (BAG_{2wk}) and clinical scores at different timepoints (n = 464 patients). Each datapoint represents one patient. p-values and R^2 are shown in each panel. Full linear models included BAG_{2wk}, sex, age, and education; F-tests compared models with and without BAG_{2wk}. Trail Making Test Part A and Part B scores were log transformed.*

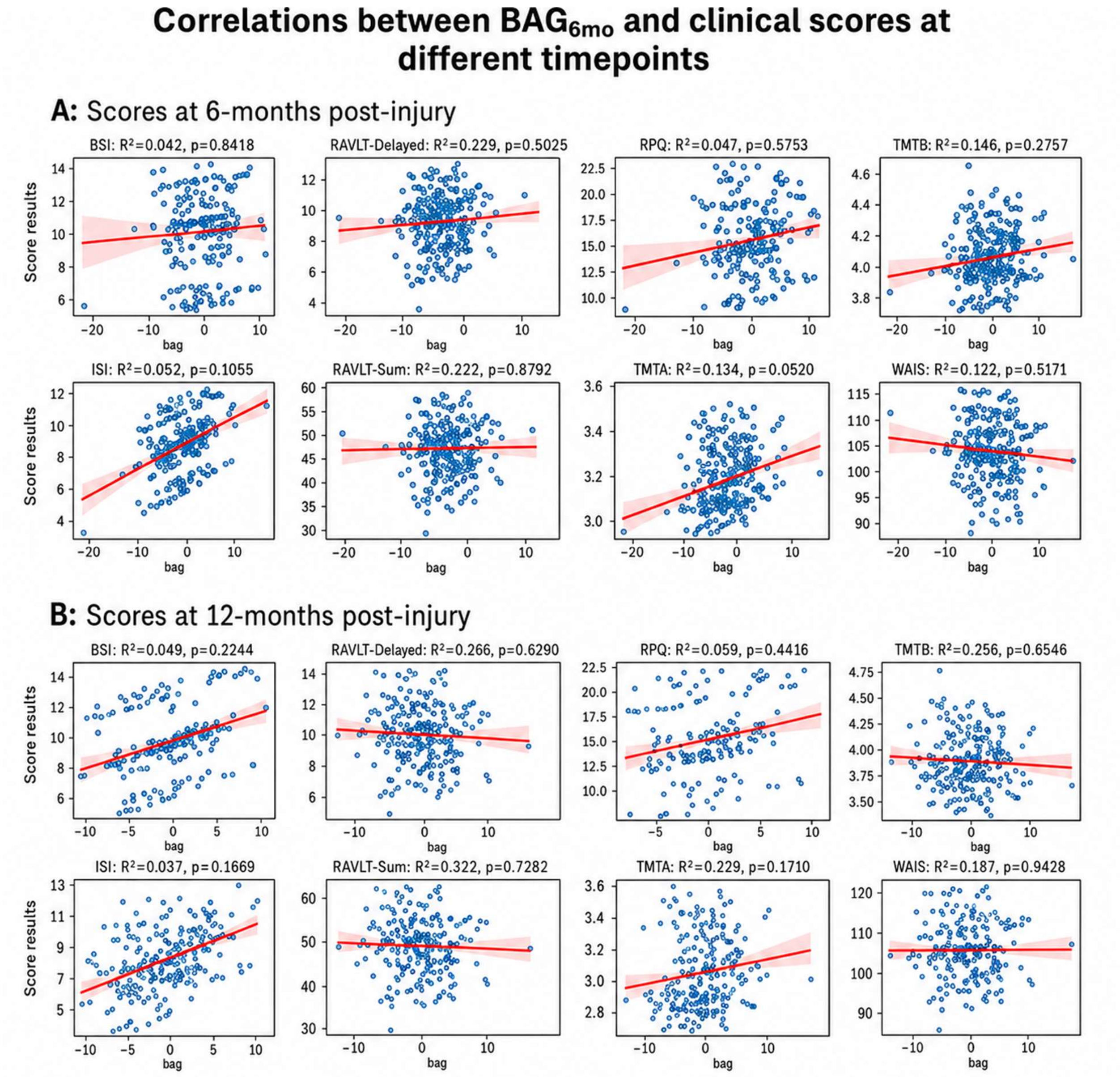

Supplementary Figure 8: *Correlations between Brain Age Gap at 6 months (BAG_{6mo}) and clinical scores at different timepoints (n = 464 patients). Each datapoint represents one patient. p-values and R^2 are shown in each panel. Full linear models included BAG_{6mo}, sex, age, and education; F-tests compared models with and without BAG_{6mo}. Trail Making Test Part A and Part B scores were log transformed.*

S.7.2 Associations between BAG and Longitudinal Trajectories of Clinical Scores

Supplementary Table 13: *Longitudinal analysis in clinical scores. Significant findings are in bold. *P-values corrected using FDR*

Score	p-value* RM vs MM	p-value* MM vs IM	p-value* RM vs IM	Partial R^2 (BAG - MM)	Partial R^2 (BAG*TSI – IM)	β [95%CI]
**BSI-18**	0.0691	0.5153	0.0760	0.0064	0.0696	0.098 [0.011, 0.180]
**ISI**	**0.0196**	0.6952	**0.0447**	**0.0104**	0.0380	**0.107 [0.033, 0.181]**
**RAVLT-Delayed**	0.5624	0.7324	0.6394	0.0011	0.0087	-0.014 [-0.050,0.018]
**RAVLT-Immediate**	0.5722	0.5153	0.6394	0.0006	0.0161	-0.005 [-0.024, 0.013]
**RPQ**	**0.0016**	0.5153	**0.0055**	**0.0174**	0.0713	**0.195 [0.093, 0.297]**
**TMTA**	0.1237	**0.0160**	**0.0059**	**0.0050**	**0.0433**	**0.028 [-0.001, 0.057]**
**TMTB**	0.5722	0.0604	0.0760	0.0007	0.0233	0.010 [-0.024, 0.045]
**WAIS**	0.5624	0.3970	0.3407	0.0012	0.0618	-0.005 [-0.018, 0.007]

When examining interactions between time since injury and demographic factors (Supplementary Figure 9), we found that education level had a significant effect on RPQ: over time, individuals with more years of education showed greater improvement compared to those with fewer years of education (p=0.0002, R^2 = 8.69%). Additionally, the interaction with sex revealed that women improved more than men over time on both RAVLT-Delayed and RAVLT-Immediate scores (p = 0.0028, R^2 = 6.26%; p= 0.0083, R^2 = 9.94%, respectively).

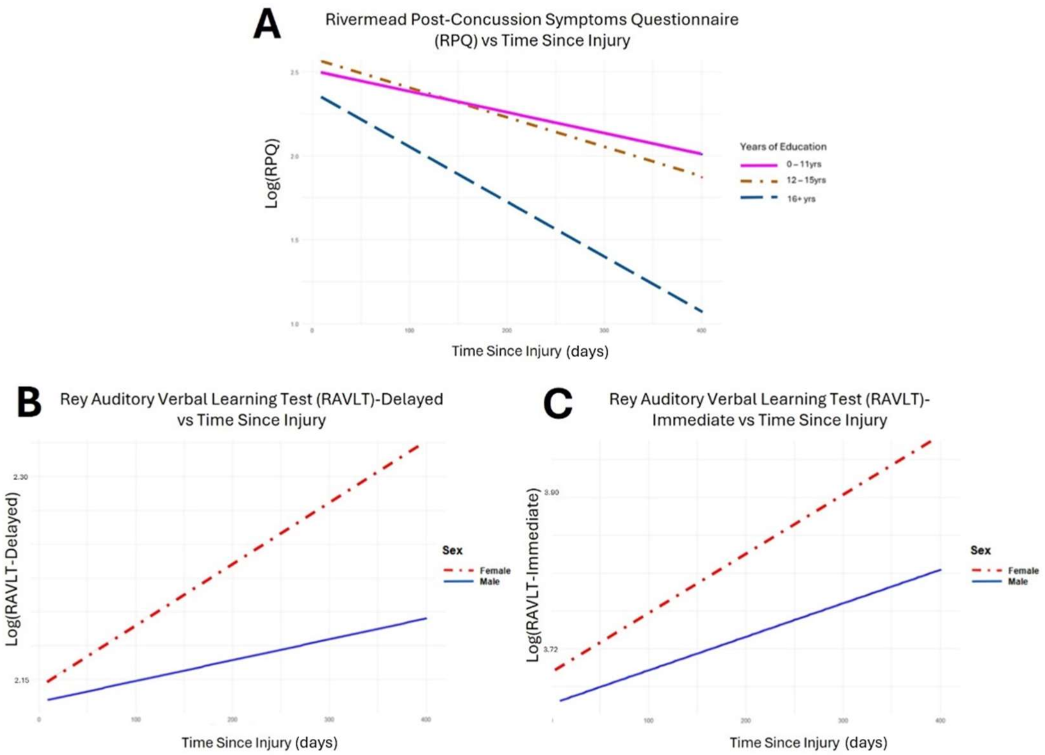

Supplementary Figure 9*: Model-predicted trajectories of Rivermead Post-Concussion Symptoms Questionnaire (RPQ) score over time based on Education Level (A) and Rey Auditory Verbal Learning Test (RAVLT) Delayed/Immediate based on Sex (B and C, respectively). Time since injury (days) is shown on the X-axis, and logged clinical scores on the Y-axis. Individuals with more years of education showed greater improvement in RPQ scores over time compared with those with fewer years of education (Likelihood-ratio test, p = 0.0002, R^2 = 8.69%). In addition, a sex-by-time interaction was observed for memory performance, with women showing greater improvement over time than men on both RAVLT-Delayed (Likelihood-ratio test, p = 0.0028, R^2 = 6.26%) and RAVLT-Immediate scores (Likelihood-ratio test, p = 0.0083, R^2 = 9.94%). Models were trained with 192 patients*

S.7.3 BAG as a marker of poor 12-months outcome

Supplementary Table 14: *Incremental value of BAG for different outcome definitions and self-reported symptoms. BAG showed a significant incremental effect only for Definition 2, with modest improvements in discrimination and model fit, while no significant effects were observed for Definitions 1, 3, or self-reported outcomes.*

**Outcome Definition**	**LRT (p)**	**ΔAUC**	**ΔMcFadden R^2**	**BAG OR [95% CI]**
**Definition 1 (cognitive)**	0.854 (0.355)	0.018	0.005	1.043 [0.963–1.128]
**Definition 2 (cognitive)**	**6.398 (0.011)**	**0.062**	**0.033**	**1.095 [1.015–1.181]**
**Definition 3 (cognitive)**	0.979 (0.322)	0.006	0.004	1.035 [0.967–1.108]
**Self-reported symptoms**	3.295 (0.070)	0.044	0.011	1.060 [0.995–1.130]

We performed an exploratory analysis to better understand which scores were more sensitive to prediction using BAG. For this, we used binary outputs indicating whether the patient showed impairment or decline for a given score (Supplementary Table 15). As before, we compared logistic regression models (without interactions).

Supplementary Table 15: Likelihood ratio tests and p-values from likelihood ratio tests comparing the full and reduced logistic regression models for each score individually.

Score	Impairment (p, LRT)	Decline (p, LRT)
**RAVLT-Delayed**	0.15, 2.11	0.10, 2.61
**RAVLT-IMMEDIATE**	0.58, 0.31	0.49, 0.47
**TMTA**	0.07, 3.29	0.05, 3.73
**TMTB**	0.74, 0.11	**0.005, 7.73**
**WAIS**	0.06, 3.5	1.0, -1.23
**ISI**	**p<0.001, 28.95**	0.08, 3.00
**BSI**	1.0, -9.95	**p<0.001, 21.49**
**RPQ**	1.0, -16.68	0.06, 3.45

Finally, we further tested whether the predictive effect of BAG_{2wk} on Definition 2 of poor cognitive outcome was modified by interacting with sex, age, or education level. No significant interactions were observed: BAG × Sex (LRT = 1.898, p = 0.168), BAG × Age (LRT = 0.373, p = 0.541), and BAG × Education (LRT = -0.029, p = 1). Inclusion of these terms did not meaningfully change discrimination (ΔAUC ≤ 0.001) or model fit (ΔMcFadden $R^2 \leq 0.010$), indicating that the effect of BAG_{2wk} was consistent across these subgroups.

References

1. Littlejohns TJ, Sudlow C, Allen NE, et al. The UK Biobank imaging enhancement of 100,000 participants: rationale, data collection, management and future directions. Nat Commun. 2020;11(1):2624. doi:10.1038/s41467-020-15948-9
2. Rohlfing T, Zahr NM, Sullivan EV, Pfefferbaum A. The SRI24 multichannel atlas of normal adult human brain structure. Hum Brain Mapp. 2010;31(5):798-819. doi:10.1002/hbm.20906
3. Avants BB, Tustison N, Song G. Advanced normalization tools (ANTS). Insight J. 2009;2(365):1-35.
4. Thakur S, Meier R, Christodoulou AG, et al. Brain extraction on MRI scans in presence of diffuse glioma: multi-institutional performance evaluation of deep learning methods and robust modality-agnostic training. Neuroimage. 2020;220:117081. doi:10.1016/j.neuroimage.2020.117081
5. Manjón JV, Coupé P, Concha L, Buades A, Collins DL. Diffusion weighted image denoising using overcomplete local PCA. PLoS One. 2013;8(9):e73021. doi:10.1371/journal.pone.0073021
6. Andersson JLR, Sotiropoulos SN. An integrated approach to correction for off-resonance effects and subject movement in diffusion MR imaging. Neuroimage. 2016;125:1063-1078. doi:10.1016/j.neuroimage.2015.10.019
7. Smith SM. Fast robust automated brain extraction. Hum Brain Mapp. 2002;17(3):143-155. doi:10.1002/hbm.10062
8. Garyfallidis E, Brett M, Amirbekian B, et al. Dipy, a library for the analysis of diffusion MRI data. Front Neuroinform. 2014;8:8. doi:10.3389/fninf.2014.00008

9. Avants BB, Tustison N, Song G, et al. Symmetric diffeomorphic image registration with cross-correlation: evaluating automated labeling of elderly and neurodegenerative brain. Med Image Anal. 2008;12(1):26-41. doi:10.1016/j.media.2007.06.004
10. Maximov II, Alnæs D, Westlye LT. Towards an optimised processing pipeline for diffusion magnetic resonance imaging data: effects of artefact corrections on diffusion metrics and their age associations in UK Biobank. Hum Brain Mapp. 2019;40(14):4146-4162. doi:10.1002/hbm.24691
11. Alfaro-Almagro F, Jenkinson M, Bangerter NK, et al. Image processing and quality control for the first 10,000 brain imaging datasets from UK Biobank. Neuroimage. 2018;166:400-424. doi:10.1016/j.neuroimage.2017.10.034
12. Andersson JLR, Skare S, Ashburner J. How to correct susceptibility distortions in spin-echo echo-planar images: application to diffusion tensor imaging. Neuroimage. 2003;20(2):870-888. doi:10.1016/S1053-8119(03)00336-7
13. Oishi K, Faria AV, Mori S. JHU-MNI-ss atlas. Johns Hopkins University School of Medicine, Department of Radiology, Center for Brain Imaging Science; 2010.
14. Oishi K, Faria AV, van Zijl PCM, Mori S. Atlas-based whole brain white matter analysis using large deformation diffeomorphic metric mapping: application to normal elderly and Alzheimer's disease participants. Neuroimage. 2009;46(2):486-499. doi:10.1016/j.neuroimage.2009.01.002
15. Fortin JP, Cullen N, Sheline YI, et al. Harmonization of multi-site diffusion tensor imaging data. Neuroimage. 2017;161:149-170. doi:10.1016/j.neuroimage.2017.08.047
16. Gan S, Shi W, Wang S et al. Accelerated brain aging in mild traumatic brain injury: longitudinal pattern recognition with white matter integrity. J Neurotrauma. 2021;38(18):2549-2559.
